# Phosphoproteomics Reveals Regulatory T Cell-Mediated DEF6 Dephosphorylation That Affects Cytokine Expression in Human Conventional T Cells

**DOI:** 10.3389/fimmu.2017.01163

**Published:** 2017-09-25

**Authors:** Rubin N. Joshi, Nadine A. Binai, Francesco Marabita, Zhenhua Sui, Amnon Altman, Albert J. R. Heck, Jesper Tegnér, Angelika Schmidt

**Affiliations:** ^1^Unit of Computational Medicine, Center for Molecular Medicine, Department of Medicine Solna, Karolinska University Hospital, Science for Life Laboratory, Karolinska Institutet, Stockholm, Sweden; ^2^Biomolecular Mass Spectrometry and Proteomics, Bijvoet Center for Biomolecular Research, Utrecht Institute for Pharmaceutical Sciences, Utrecht University, Utrecht, Netherlands; ^3^Netherlands Proteomics Centre, Utrecht, Netherlands; ^4^Division of Cell Biology, La Jolla Institute for Allergy and Immunology, La Jolla, CA, United States; ^5^Biological and Environmental Sciences and Engineering Division, King Abdullah University of Science and Technology (KAUST), Thuwal, Saudi Arabia; ^6^Computer, Electrical and Mathematical Sciences and Engineering Division, King Abdullah University of Science and Technology (KAUST), Thuwal, Saudi Arabia

**Keywords:** regulatory T cell, Treg, CD4 T cell, phosphoproteomics, DEF6, SLAT, NFAT, TCR signaling

## Abstract

Regulatory T cells (Tregs) control key events of immune tolerance, primarily by suppression of effector T cells. We previously revealed that Tregs rapidly suppress T cell receptor (TCR)-induced calcium store depletion in conventional CD4^+^CD25^−^ T cells (Tcons) independently of IP_3_ levels, consequently inhibiting NFAT signaling and effector cytokine expression. Here, we study Treg suppression mechanisms through unbiased phosphoproteomics of primary human Tcons upon TCR stimulation and Treg-mediated suppression, respectively. Tregs induced a state of overall decreased phosphorylation as opposed to TCR stimulation. We discovered novel phosphosites (T595_S597) in the DEF6 (SLAT) protein that were phosphorylated upon TCR stimulation and conversely dephosphorylated upon coculture with Tregs. Mutation of these DEF6 phosphosites abrogated interaction of DEF6 with the IP_3_ receptor and affected NFAT activation and cytokine transcription in primary Tcons. This novel mechanism and phosphoproteomics data resource may aid in modifying sensitivity of Tcons to Treg-mediated suppression in autoimmune disease or cancer.

## Introduction

Regulatory T cells (Tregs) play an indispensable role in the immune system, being critical mediators of peripheral self-tolerance by suppression of effector T cells and other immune cells. The majority of human Tregs in the peripheral blood are represented within the CD4^+^CD25^++^ population that highly expresses the Treg lineage-defining transcription factor Forkhead box P3 (FOXP3) (1). Due to lack of a unique protein surface marker designating Tregs, the ultimate way to define Tregs is by their suppressive function. Their immune-suppressive properties are necessary to prevent autoimmune disease, allergies, and infection-induced pathology. However, Tregs can also dampen immune responses against pathogens and tumors in certain settings (2). First-in-man trials on adoptive Treg transfer and therapeutic targeting of Tregs are ongoing, for example, in human autoimmune disease (type 1 diabetes), graft-versus-host disease, and cancer with partially good results (2–5). Notwithstanding this recent progress in manipulating Tregs, for several autoimmune diseases, it has been reported that there is no defect in Treg number or function, but rather resistance of responder T cells to suppression (6, 7), underlining the significance of research on suppressive mechanisms and events in the target cells.

Tregs suppress proliferation and cytokine expression of conventional CD4^+^CD25^−^ T cells (Tcons) by diverse mechanisms, which may operate depending on the site and cytokine milieu of the immune reaction as well as on subtype and activation status of target cells and Tregs (8, 9). These Treg suppression mechanisms include release of immune-suppressive cytokines, competition for survival factors, apoptosis induction, and indirect inhibition of Tcons through suppression of antigen-presenting cells (APCs), the latter being largely mediated by cytotoxic T lymphocyte-associated protein 4 (CTLA-4) that is constitutively expressed in Tregs. Another hallmark of Tregs is their ability to directly suppress Tcons in a contact-dependent manner *in vitro* (10). While early studies imaging Tregs in intact explanted or intravital lymph nodes concluded that stable direct contacts of Tregs with Tcons do not occur *in vivo* (11, 12), a recent breakthrough study (13) discovered that at the site of inflammation in non-lymphoid target tissues, Tregs stably contact conventional effector T cells. This study (13) of pancreatic autoimmune-induced damage and graft rejection also demonstrated that Treg:Tcon interaction occurred with or without engagement of APCs, and CTLA-4 had only a marginal role. Additional studies confirmed direct Treg:Tcon contacts *in vivo* in lymph nodes, in this case in an antigen-specific and CTLA-4-dependent manner (14). Since Tregs and Tcons directly interact *in vivo* and *in vitro*, and Tcons as one of the main Treg target cells drive several autoimmune diseases and are involved in tumor clearance, understanding their inhibition is of major importance. Yet, only few data are available on signaling events in suppressed Tcons upon contact to Tregs.

Triggering the T cell receptor (TCR) in the presence of costimulatory (CD28-engaging) signals leads to a cascade of protein phosphorylation and dephosphorylation events in Tcons, which ultimately activates transcription factors to initiate a distinct transcriptional program. Besides others, key transcription factors mediating TCR-induced cytokine expression in Tcons are NFAT, AP-1, and NF-κB (8, 15). TCR-induced signaling pathways are very well understood and involve a multitude of signaling molecules. Remodeling of the cytoskeleton is involved in TCR signaling, primarily affecting NFAT pathways (16, 17). Cytoskeleton rearrangement, besides enabling the clustering and polarization of the TCR signalosome, aids in triggering the elevation of intracellular calcium (Ca^2+^) levels (17, 18), which is the major event activating NFAT in T cells: TCR stimulation leads to phosphorylation and activation of the lipase PLC-γ, which cleaves phosphatidylinositol 4,5-bisphosphate (PIP_2_) into the second messengers diacylglycerol (DAG) and inositol 1,4,5-trisphosphate (IP_3_). IP_3_ in turn binds to IP_3_ receptors (IP_3_Rs) in the endoplasmic reticulum (ER) membrane, releasing Ca^2+^ from the ER into the cytoplasm. ER Ca^2+^ store depletion is sensed by the stromal interaction molecules (STIMs), which then trigger opening of calcium release-activated calcium (CRAC) channels in the plasma membrane and influx of extracellular Ca^2+^ into the cell. This so-called store-operated calcium entry (SOCE) is the major pathway leading to Ca^2+^ signals in T cells although other second messengers and channels are also involved (19, 20). Cytoskeleton remodeling promotes both phases leading to SOCE (17). For example, the actin remodeling complex WAVE is crucial for CRAC channel opening (21). Another important player involved here is the protein DEF6 (differentially expressed in FDCP 6 homolog), also known as SWAP-70-like adaptor of T cells (SLAT) (16). DEF6 is phosphorylated by Lck after TCR stimulation and is required for intracellular Ca^2+^ store depletion, NFAT activation, and IL-2 production (16, 22), interestingly in an IP_3_-independent manner and directly interacting with the IP_3_R (23). Ca^2+^ activates the phosphatase calcineurin which subsequently dephosphorylates NFAT, thereby unmasking its nuclear localization sequence and enabling NFAT translocation to the nucleus.

Although TCR signaling *per se* is well understood, only few studies have addressed TCR signaling in T cells during their suppression by Tregs. Our previous study in human Tcons revealed that Tregs directly and rapidly suppress TCR-induced Ca^2+^, NFAT, and NF-κB activation in target Tcons and consequently IL-2 and IFN-γ cytokine expression, while TCR-proximal and AP-1 signals were unaffected (24). The most upstream suppressed event was Ca^2+^ store depletion independently of IP_3_ levels (24). Notably, Schwarz et al. subsequently confirmed Treg-mediated Ca^2+^ suppression in another experimental setup and revealed an impairment of such suppression in multiple sclerosis patients (25). Others followed up studying individual signaling molecules in Treg-suppressed Tcons of human or murine origin under diverse experimental conditions (26–28). However, none of these publications goes beyond the study of well-known TCR signaling molecules. So far unknown molecules initiating suppression may be revealed by global unbiased studies of signaling events in Treg-suppressed Tcons, which are lacking to date.

Due to the short time period (within 30 min of coculture) required to induce suppression (24), we hypothesized that Tregs may provoke rapid post-translational modifications (PTMs), such as (de)phosphorylations, in suppressed Tcons. Thus, we here performed an unbiased, quantitative state-of-the-art mass spectrometry (MS)-based phosphoproteomic analysis of primary human Tcons in the unstimulated, stimulated, and Treg-suppressed stimulated states. We show that TCR stimulation led to generally enhanced protein phosphorylation that was counteracted by Tregs. Importantly, Tregs reduced phosphorylation of DEF6 in suppressed Tcons, which occurred at yet uncharacterized phosphosites: threonine 595 (T595) and serine 597 (S597). Mutation of these phosphosites confirmed their importance in DEF6:IP_3_R interaction, NFAT activation, and IL-2 and IFN-γ cytokine expression in cell lines and primary T cells, respectively. In line with our previous results that Tregs rapidly suppress Ca^2+^ store depletion without affecting IP_3_ levels (24), we propose a novel suppression mechanism in which Tregs cause DEF6 dephosphorylation, thus preventing DEF6 interaction with the IP_3_R and consequently cytokine transcription in suppressed Tcons. Our phosphoproteomics data are a valuable resource of signaling events in Tcons upon TCR stimulation and Treg-mediated suppression, advancing basic knowledge on these fundamental immunological processes, and for the first time linking DEF6 to Treg-mediated suppression. Although future studies have to address the functional relevance of these results *in vivo* in the context of T cell activation and suppression, the results may have important implications for therapeutic manipulation of Treg-mediated suppression in the future. In cancer, suppression of effector T cells can be deleterious and breaking suppression is desirable, while during autoimmunity, a suppressed state of autoreactive T cells is warranted. Signaling in suppressed Tcons is particularly relevant in light of the findings that direct Treg:Tcon interactions occur *in vivo* at the inflammatory site, and that effector T cells are frequently resistant to Treg-mediated suppression in human autoimmune disease.

## Materials and Methods

### Ethics Statement

Human peripheral blood mononuclear cells (PBMCs) were freshly isolated from anonymized healthy donor buffy coats purchased from the Karolinska University Hospital (Karolinska Universitetssjukhuset, Huddinge), Sweden. Research was performed according to the national Swedish ethical regulations (ethical review act, SFS number 2003:460). Ethical permit for the experiments was obtained from the Regional Ethical Review Board in Stockholm (Regionala etikprövningsnämnden i Stockholm), Sweden (approval number: 2013/1458-31/1).

### Isolation of Human Tregs and Tcons

Human peripheral blood leukocytes were purified from fresh buffy coats by gradient centrifugation using Ficoll-Paque Plus (GE Healthcare), followed by plastic adherence in RPMI 1640 medium containing 10% FCS (Invitrogen) to deplete monocytes. Blood from HLA-A2^+^ donors was used to isolate Tregs and Tcons, and blood from HLA-A2^−^ donors was used to isolate responder Tcons. Before Treg isolation, PBMCs were rested overnight at 4°C, or directly used for magnetic-activated cell sorting (MACS) isolation. We first isolated CD25^high^ cells with CD25-specific MACS beads (2 μl/10^7^ cells, Miltenyi Biotec; cat. no. 130-092-983) as described previously (24). “Untouched” CD4^+^CD25^−^ Tcons were isolated using the CD4^+^ T cell Isolation Kit, human (Miltenyi Biotec) and were additionally depleted from CD25^+^ cells with CD25-specific MACS beads (8 μl/10^7^ cells). Cell purity of all MACS-isolated cells was assessed using flow cytometry. Cells were counted in trypan blue solution using the Countess Automated Cell Counter (Life Technologies), and viability was determined using trypan blue stain and/or flow cytometry (see below). Tcons and Tregs were cultured at 5% CO_2_/37°C in serum-free X-Vivo 15 medium (Lonza) containing 1% GlutaMAX (Invitrogen).

### Pre-Activation of Tregs

Tregs were pre-activated overnight with covalently plate-bound antibody against CD3 as described (24), to prevent potential carryover of pre-activating antibody bound to Tregs into cocultures. In brief, amine-binding, maleic anhydride-activated 96F well plates (Pierce) were washed with wash buffer [PBS, 0.05% (v/v) Tween-20] and with PBS before incubation with 10 μg/ml anti-CD3 antibody (100 μl/well in PBS; clone OKT3; Biolegend, LEAF grade) for 3 h at 20°C. Wells were then washed with wash buffer and PBS and blocked with X-VIVO 15 medium for 2.5 h at 20°C. Plates were then washed with wash buffer and with X-VIVO 15 medium, and the medium was completely removed. Tregs were incubated on the plates at a density of 2–3 × 10^6^ cells/ml (200 μl/well) in X-VIVO 15 medium containing 1% GlutaMAX and IL-2 (100 IU/ml; R&D Systems) overnight at 37°C/5% CO_2_.

### Coculture Setup and T Cell Stimulation

HLA-A2^+^ Tregs (pre-activated and subsequently pooled from three to five donors to obtain sufficient cell numbers) and HLA-A2^+^ Tcons (control) were labeled with fluorescein isothiocyanate (FITC)-conjugated antibody against HLA-A2, and FITC-specific microbeads (Miltenyi Biotec), whereas HLA-A2^−^ Tcons (responder Tcons) were left untreated. Before setup of cocultures and stimulation, all cells were thoroughly washed in X-VIVO 15 medium and resuspended in X-VIVO 15 medium. Cocultures of HLA-A2^−^ responder Tcons (one donor per experiment) with either HLA-A2^+^ Tregs or control HLA-A2^+^ Tcons were set up in a 1:1 ratio. Cells were cocultured for 85 min and then stimulated. T cells were stimulated with soluble antibody against CD3 (0.2 μg/ml, clone OKT3, Biolegend, LEAF grade, cat. no. 317315), antibody against CD28 (2 μg/ml, clone 15E8, Miltenyi Biotec, functional grade, cat. no. 130-093-375), and goat anti-mouse Ig antibody as a cross-linker (2 μg/ml, SouthernBiotech, cat. no. 1010-01) mimicking TCR and costimulation. Cells were stimulated for 5 min for protein studies and for 3–4 h for RNA studies, respectively, at 37°C and 5% CO_2_. As controls, HLA-A2^−^ Tcons were left unstimulated or stimulated alone (without allogeneic cell coculture, at the same final cell density and number). For validation experiments, Tcons from single donors (without cocultures) were stimulated in the same way. Stimulation was then stopped with ice-cold MACS buffer [0.5% (w/v) human serum albumin, 2 mM EDTA, in PBS], and where applicable the different cell populations were separated on the basis of HLA-A2 expression by passing the cells over an LS column (Miltenyi Biotec) on ice; control cells including unstimulated cells were treated and passed over LS columns in the same way. HLA-A2^−^ Tcons (flow through from the column; 97 ± 3% pure, mean ± SD; individual values, see Figure S1D in Supplementary Material) were used for subsequent phosphoproteomics and mRNA analyses. For phosphoproteomics, 8–12 × 10^6^ responder Tcons per sample were used. An aliquot of cultures from each phosphoproteomic experiment was stimulated for 3 h before coculture separation to assess the responsiveness of responder Tcons to stimulation and Treg-mediated suppression by analyzing the abundances of *IL2* and *IFNG* mRNAs with quantitative RT-PCR (qRT-PCR) in the HLA-A2^−^ Tcons. After stimulation (and coculture separation where applicable), cells were centrifuged (450 × *g*, 8 min, 4°C) and supernatant was removed. For protein studies, cells were further washed twice with 1 ml ice-cold PBS each (1,000 × *g*, 5 min, 4°C) and the supernatant was removed completely before use of cell pellets (see below). For RNA studies, cell pellets were stored at −20°C before analysis (see below).

### RNA Preparation and qRT-PCR

Total RNA was isolated using the RNAqueous Micro Kit (Ambion), quantified using the Nanodrop 2000 (Thermo Scientific), and cDNA was prepared using the SuperScript VILO cDNA Synthesis Kit (Invitrogen) according to the manufacturer’s instructions. mRNA was quantified using Taqman probes (Applied Biosystems best coverage probes for *DEF6, RPL13A, IFNG*, and *IL2*; FAM reporter) with the Taqman gene expression master mix (Applied Biosystems) or with SYBR Green primers (Sigma-Aldrich) with the Power SYBR Green PCR Master Mix (Applied Biosystems) and measured on a StepOne plus detector system (Applied Biosystems). The relative mRNA expression was determined by normalization to *RPL13A* and/or *GAPDH*. Results are presented as fold induction compared to mRNA amounts of unstimulated Tcons of the same donor, which were set to 1. Fold expression was calculated using the ΔΔ*C*_t_ method according to the following formula (*C*_t_ is the threshold cycle value):

$$\text{Relative mRNA expression}=2^{–({\text{C}}_{\text{t}}\;\text{of gene of interest}–{\text{C}}_{\text{t}}\;\text{of RPL}13\text{A})}$$

SYBR Green primer sequences were as follows: *GAPDH*: 5′-GCA AAT TCC ATG GCA CCG T-3′ (forward) and 5′-TCG CCC CAC TTG ATT TTG G-3′ (reverse); *IL2*: 5′-CAA CTG GAG CAT TTA CTG CTG G-3′ (forward) and 5′-TCA GTT CTG TGG CCT TCT TGG-3′ (reverse); *IFNG*: 5′-TTC AGC TCT GCA TCG TTT TGG-3′ (forward) and 5′-TCC GCT ACA TCT GAA TGA CCTG-3′ (reverse); *IL2RA*: 5′-CAC TCG GAA CAC AAC GAA ACA-3′ (forward) and 5′-TGT GGC TTC ATT TTC CCA TG-3′ (reverse); *FASLG*: 5′-TGG AAT TGT CCT GCT TTC TGG-3′ (forward) and 5′-TGT TGC AAG ATT GAC CCC G-3′ (reverse); *RPL13A*: 5′-TCC AAG CGG CTG CCG AAG ATG-3′ (forward) and 5′-CTT CCG GCC CAG CAG TAC CTGT-3′ (reverse).

### Phosphoproteomics

#### Sample Preparation

Responder Tcons were prepared as described earlier, and cell pellets were snap-frozen in liquid N_2_ and stored at −80°C until use. Cells were lysed in lysis buffer [8 M urea, 1 mM sodium orthovanadate, 1× phosSTOP (Roche), 1× protease inhibitor tablets (Roche) in 100 mM Tris, pH 8.5]. Cells were homogenized by vortexing for 10 min. Lysates were cleared by centrifugation (10 min, 20,000 × *g*, 4°C). Supernatant was transferred into a new tube, and the protein amount was determined using the Bio-Rad Protein assay (Bio-Rad). Next, 57–196 μg protein per sample (135 ± 54 μg; Donor 1: 57–99 μg; Donor 2: 173–196 μg; Donor 3: 119–191 μg) was digested utilizing the filter-assisted sample preparation protocol (29). The reduced and alkylated sample was first treated with Lys-C (Sigma-Aldrich) in an enzyme:substrate ratio of 1:50 (w/w) for 4 h, after this trypsin (Promega) was used overnight at 37°C in an enzyme:substrate ratio of 1:100 (w/w). Resulting peptide mixtures were desalted and chemically labeled using stable isotope dimethyl labeling, as described previously (30). The unstimulated Tcon (“Trest”) sample was labeled “light,” the stimulated Tcons (“Tstim”) were labeled “medium” and the “heavy” label was used for the stimulated suppressed Tcon sample (“Tsup”). The samples were mixed in a 1:1:1 (L:M:H) ratio based on peptide intensities.

#### Phosphopeptide Enrichment by Ti^4+^-IMAC

Ti^4+^-IMAC material was prepared and used as described earlier (31). Briefly, 500 μg Ti^4+^-IMAC beads were loaded onto a GELloader tip (Eppendorf) with a C8 plug. After conditioning the columns with loading buffer [80% acetonitrile (ACN), 6% trifluoroacetic acid], samples reconstituted in loading buffer were loaded onto the columns and centrifuged at 100 × *g* for 30 min. In total, four washing steps were performed to ensure the removal of unbound peptides from the columns. The bound peptides were eluted into a new tube containing 30 μl 10% formic acid (FA) with 20 μl 10% ammonia. A final elution was performed with 2 μl 80% ACN/2% FA. The eluate was further acidified by adding 3 μl of 100% FA and directly used for liquid chromatography (LC)-MS analysis.

#### LC-MS

The phospho-enriched samples were directly analyzed on an Orbitrap Elite mass spectrometer coupled to an Easy UHPLC system (both Thermo Fisher Scientific) during a 2-h gradient (7–30% ACN 91 min, 30–100% ACN 3 min, 100% ACN 5 min, 100–7% 1 min, 7% ACN 20 min, and flow rate: 100 nl/min). The Elite mass spectrometer was operated in data-dependent acquisition mode using the following settings: ESI voltage, 1.5 kV; inlet capillary temperature, 320°C; full scan automatic gain control (AGC) target, 1e6 ions at 60,000 resolution; scan range, 350–1,500 *m*/*z*; Orbitrap full scan maximum injection time, 250 ms; data-dependent decision tree (HCD/ETD) (32); normalized collision energy, 32; dynamic exclusion time, 30 s; isolation window, 1.5 *m*/*z*; 20 MS2 scans per full scan.

#### MS Data Analysis

The raw data obtained were initially processed with Proteome Discoverer (PD) 1.4 (Thermo Fisher Scientific). The created peak lists were searched with Mascot (Matrix Science, Version 2.3) against a concatenated forward–reverse Uniprot database (taxonomy *Homo sapiens*, containing 41,008 entries) and the following parameters: 50 ppm precursor mass tolerance and 0.05 Da fragment ion tolerance for OT spectra and 0.6 Da fragment ion tolerance for IT spectra. Up to two missed cleavages were accepted, oxidation of methionine, phosphorylation of STY, and the dimethyl label on lysines and the N-terminus was set up as variable modification whereas cysteine carbamidomethylation as fixed modifications. For the phospho-enriched samples, the site occupation probabilities were calculated using the phosphoRS node in PD. Afterward, all phosphosites were filtered for 75% localization probability. Triplex dimethyl labeling was chosen as quantification method. All peptides were filtered for a minimal Mascot score of 20 and 1% FDR.

### Differential Phosphorylation Statistical Analysis

Data preprocessing, normalization, and differential phosphorylation analyses were performed in R. Unique phosphopeptides were summarized from the phosphoRS output, by grouping peptides: (1) mapping to the same protein Uniprot accession, (2) having the same sequences, and (3) having the same localized phosphosites. The corresponding L (light), M (medium), and H (heavy) intensities from Trest, Tstim, and Tsup were summarized by averaging multiple occurrences of the same phosphopeptide. Uniprot to gene symbol and Entrez ID conversion was obtained from the org.Hs.eg.db library. Only phosphopeptides detected in at least two out of three donors were considered for further analysis. Between-sample quantile normalization was performed on the pooled raw signal intensities, assuming that the intensity distribution of the pooled L, M, and H channels are similar for different samples, and the limma function normalizeQuantiles performed the normalization routine. Intensity values were log_2_-transformed and missing values were imputed using nearest neighbor averaging from the impute library (33). The resulting matrix with imputed values was used to fit a linear model in limma with the group (L for Trest, M for Tstim, and H for Tsup) and the donor as factors in the design matrix. The contrasts to study the TCR stimulation-induced and the suppression-induced changes were extracted (i.e., Tstim:Trest, Tsup:Tstim, and Tsup:Trest) and then Empirical Bayes moderated *t* test was performed and *P* values were calculated with limma.

### Expression Plasmids and Mutagenesis

pEGFP-N3 (Clontech) was used as a backbone (empty vector, EV). For overexpression of *DEF6*, EGFP was replaced by full-length, N-terminally myc-tagged *DEF6* cDNA (DEF6-WT). To create phospho-silent (DEF6-2A) or phospho-mimic (DEF6-2E) mutants of T595 and S597 phosphosites, both sites were replaced by alanine (A) or glutamic acid (E) residues, respectively, using the QuikChange II XL Site-Directed Mutagenesis Kit (Stratagene), with primers as follows:

2A-forward: 5′-CCAGGGCAACAGGGCCCCCGCGCCCAACAGCA-3′
2A-reverse: 5′-TGCTGTTGGGCGCGGGGGCCCTGTTGCCCTGG-3′
2E-forward: 5′-CCAGGGCAACAGGGAACCCGAGCCCAACAGCA-3′
2E-reverse: 5′-TGCTGTTGGGCTCGGGTTCCCTGTTGCCCTGG-3′.

Plasmids were transformed to NEB 10-beta competent *E. coli* (High Efficiency) (cat. no. C3019, New England Biolabs) for amplification and purified using the EndoFree Plasmid Maxi Kit (Qiagen; cat. no. 12363). DEF6-WT and mutant sequences were verified by sequencing (using CMV forward primer, middle primer located at ~700 bp, and pFastBacR reverse primer). FLAG-IP_3_R plasmid was generated as previously described (23, 34).

### Coimmunoprecipitation

HEK293 cells were tested negative for *Mycoplasma* contamination as follows: LookOut Mycoplasma PCR Detection Kit (Sigma-Aldrich, cat. no. MP0035) was used to test *Mycoplasma* contamination of HEK293 cells. Medium and FBS were tested prior to using it with the cells, and culture supernatants were tested after growing the cells for the amount of time suggested by the manufacturer. For coimmunoprecipitation of DEF6 and IP_3_R, HEK293 cells were transfected with DNA plasmids encoding Myc-DEF6 (DEF6-WT), Myc-DEF6-2A, FLAG-IP_3_R, using Lipofectamine 3000 (Life Technologies). After incubation for 48 h at 37°C, the cells were treated with 20 mM pervanadate for 5 min and lysed using radioimmunoprecipitation assay buffer [50 mM Tris, pH 7.4, 150 mM NaCl, 1% nonyl phenoxypolyethoxylethanol (NP-40), 0.02% sodium dodecyl sulfate (SDS), 1 mM phenylmethane sulfonyl fluoride, 10 μg/ml leupeptin, 5 μg/ml aprotinin, and 1/100 phosphatase inhibitor]. After centrifugation at 13,000 × *g* (10 min at 4°C), the supernatants were incubated using the EZview™ Red anti-MYC Affinity Gel (Sigma-Aldrich) overnight at 4°C. The beads were washed three times, and bound proteins were analyzed by SDS-polyacrylamide gel electrophoresis (PAGE). Proteins were detected by immunoblotting using mouse anti-FLAG (clone M2, Sigma-Aldrich, cat. no. F1804) and anti-Myc (clone 9E10, Santa Cruz Biotechnology, cat. no. SC-40) antibodies.

### Plasmid Transfection of Primary Human T Cells

5 × 10^5^ Tcons were mixed with 0.5 μg of DEF6-WT, DEF6-2A mutant, DEF6-2E mutant, or EV plasmids per electroporation at 5 × 10^7^ cells/ml and electroporated using the Neon Transfection System 10 μL Kit (Thermo Fisher Scientific) (2,400 V, 17 ms, 1 pulse) as per the manufacturer’s recommendation (multiple 96-U wells subsequently pooled where necessary). Cells were incubated in antibiotics-free RPMI 1640 medium containing 10% FCS (Invitrogen) for 8 h. Transfection parameters were optimized using 24-well optimization parameters with EV and assessed by the total fraction of GFP^+^ cells, as well as cell viability based on physical parameters of cells in flow cytometry. Amount of plasmid and duration of post-transfection incubation period were further optimized by measuring *IL2* and *IFNG* mRNA expression after 1.5, 3, and 10 h of TCR activation (data not shown).

### Western Blot

Transfected Tcons after incubation were stimulated and washed with PBS as described earlier, and lysed in Beadlyte Cell Signaling Universal Lysis Buffer (Upstate) supplemented with Halt Protease and Phosphatase Inhibitor Cocktail (Thermo Fisher Scientific, cat. no. 78440). Proteins were denatured in SDS sample buffer, resolved by SDS-PAGE using 10% Mini-PROTEAN TGX Gel (Bio-Rad, cat. no. 4561035S), and transferred to Protran nitrocellulose membranes (Amersham GE Healthcare, cat. no. 10600002). Afterward, the membranes were blocked with 5% non-fat dry milk in TBS containing 0.1% (w/v) Tween 20 (TBST) and incubated with primary (see below) and horseradish peroxidase (HRP)-conjugated secondary antibodies (Santa Cruz Biotechnology). Protein bands were developed with Immobilon Western Chemiluminescent HRP substrate (Millipore) in a Vilber Fusion Solo S chemiluminescence acquisition system (Vilber Lourmat). Bands were quantified using the ImageJ software version 1.5Oi. Anti-NFAT1 (clone 4G6-G5, Santa Cruz Biotechnology, cat. no. sc-7296), anti-myc (clone 9B11, Cell Signaling Technology, cat. no. 2276), anti-DEF6 (clone EPR7492, Abcam, cat. no. ab126792), and anti-GAPDH (clone 6C5, Santa Cruz Biotechnology, cat. no sc-32233) antibodies were used for Western blot to detect corresponding targets.

### Flow Cytometry

Staining with anti-HLA-A2-PE (clone BB7.2, BD Biosciences, cat. no. 558570), anti-HLA-A2-FITC (clone BB7.2, BD Biosciences, cat. no. 551285), anti-CD4-PerCP (clone SK3, BD Biosciences, cat. no. 345770), and/or anti-CD25-PE (clone 4E3, Miltenyi Biotec; cat. no. 130-091-024) was performed in the dark with antibody dilutions in FACS buffer (PBS/0.5% HSA) for 15 min at 20°C or 30 min at 4°C. Cells were washed once with PBS, resuspended, and measured in FACS buffer. For intracellular FOXP3 staining, cells were first stained with surface antibodies as described earlier, followed by fixable viability dye-eFlour780 (ebioscience, cat. no. 65-0865-14) staining enabling gating on live cells. Subsequently, fixation, permeabilization, and intracellular staining was performed with the Foxp3 Staining Buffer Set (ebioscience, cat. no. 00-5523-00) using anti-FOXP3-APC (clone 236A/E7, ebioscience, cat. no. 17-4777-42) or equally concentrated mIgG1κ APC isotype control (clone P3.6.2.8.1, ebioscience, cat. no. 17-4714-42) antibodies. Acquisition was performed on a CyAn ADP 9 Color Analyzer (Beckman Coulter), and parameter compensation was performed automatically with the CyAn software (Summit) tool using single stained samples containing positive cells. Flow cytometry data were analyzed using the FlowJo software (Tree Star).

### Statistical Analyses

Statistical analyses were performed in R programming language (see above) or in GraphPad Prism version 7. Statistical tests used are noted in individual figure legends and/or individual parts in the Section “Materials and Methods.”

### Accession Codes

Mass spectrometry proteomics data are deposited to the ProteomeXchange Consortium *via* PRIDE (35) partner repository; dataset identifier PXD004291 (“Mix 1/2/3” = Donor 1/2/3) and PXD006142 (Donor 4).

## Results

### A Tcon:Treg Coculture System to Study Rapid Treg-Mediated Suppression of Tcon Signaling on a Phosphoproteomic Scale

**Figure 1:**
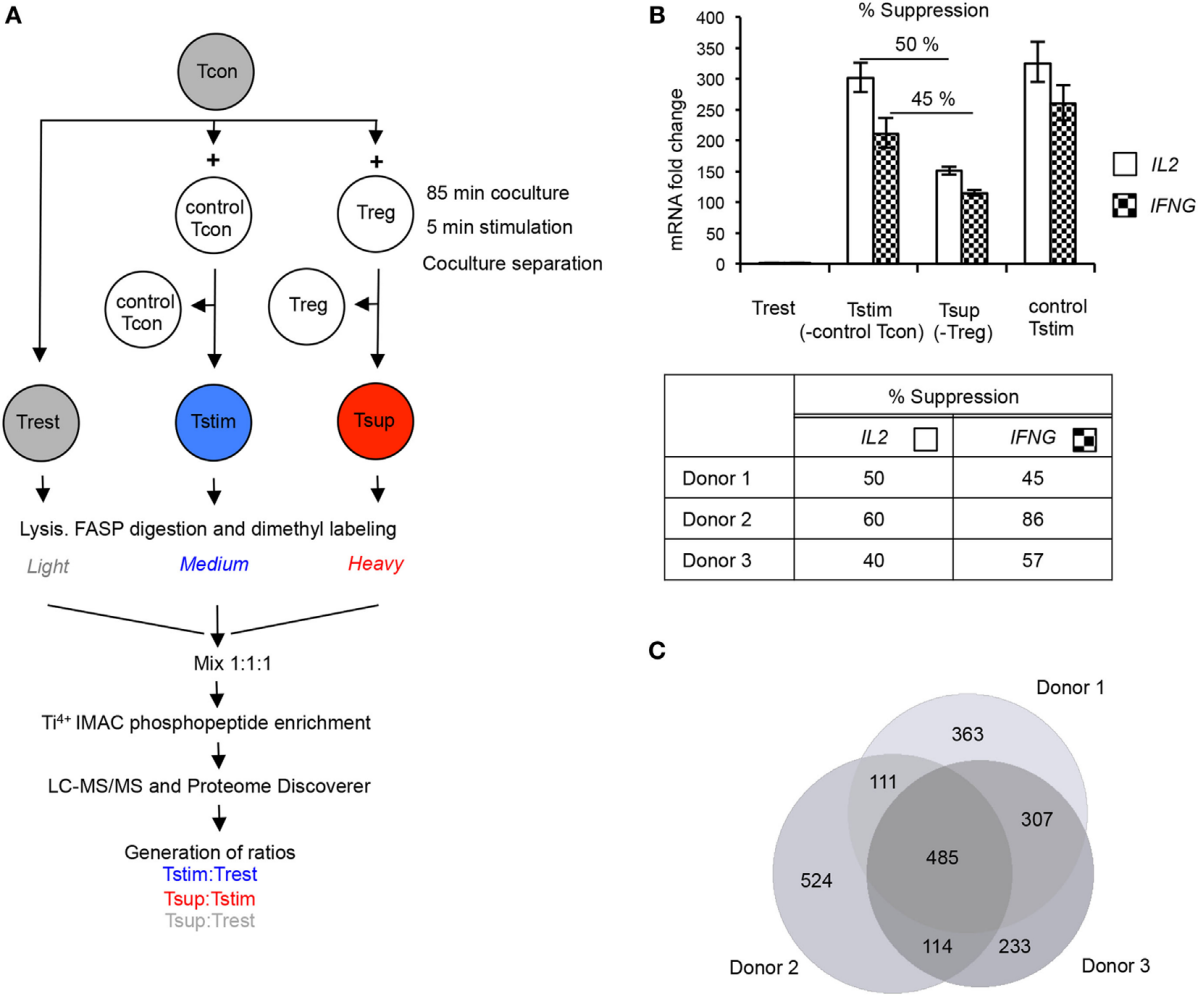
Experimental setup and quality controls for phosphoproteomics in primary human T cells. **(A)** Conventional CD4^+^CD25^–^ T cells (Tcons) were cocultured either with allogeneic Tcons or regulatory T cells (Tregs), and cocultures were stimulated for 5 min with cross-linked anti-CD3/anti-CD28 antibodies. Stimulation was stopped on ice. Tstim (blue) and Tsup (red) were obtained after separation of T cell receptor (TCR)-stimulated Tcon:Tcon or Tcon:Treg cocultures, respectively. Unstimulated Tcons (Trest; gray) from the same donor were processed in parallel. Proteins were digested, peptides dimethyl-labeled and mixed, before phosphopeptides were enriched and measured by mass spectrometry (MS). Relative abundance of phosphopeptides was quantified by calculating the intensity ratios between the different samples as indicated. **(B)** An aliquot of cells used for phosphoproteomics was stimulated for 3 h before coculture separation, and suppression of cytokine mRNA was measured in re-isolated responder Tcons [Trest, Tstim, and Tsup as in panel **(A)**]. As additional control, responder Tcons were stimulated without allogeneic Tcons (control Tstim). *IL2* and *IFNG* mRNA were measured by quantitative RT-PCR, normalized to *GAPDH* mRNA. Results are presented as fold change compared to Trest (set to 1). The upper panel shows a representative donor (mean ± SD of technical PCR duplicates). Percentage suppression of respective cytokines in Tsup as compared to Tstim was calculated and is summarized for the three phosphoproteomics donors (lower panel). T cells were processed in three independent experiments (one experiment/donor) and phosphopeptide enrichment was performed in two independent experiments. **(C)** The number of unique phosphopeptides detected in each donor was determined, and the overlap is depicted as Venn diagram.

To decipher the causative events resulting in Treg-mediated rapid suppression of Tcons and to obtain a global overview of suppression-induced molecular changes, we performed MS-based unbiased phosphoproteomic analysis of Treg-suppressed Tcons (Tsup), TCR-stimulated Tcons (Tstim), and unstimulated resting Tcons (Trest). To do so, we isolated primary Tregs and Tcons from human peripheral blood and employed a coculture system in which HLA-A2 from allogeneic cells is used as a marker to distinguish and rapidly separate the cocultured cells (24). Here, signaling can be studied in pure, re-isolated HLA-A2^−^ responder Tcons from cocultures with either HLA-A2^+^ pre-activated Tregs or HLA-A2^+^ control Tcons to attain Tsup (from coculture with Tregs) and Tstim (from coculture with Tcons) for phosphoproteomic analysis (Figure 1A). Since we were interested in deciphering causative and proximal events of suppression, we cocultured the cells for 85 min and then stimulated the cocultures with TCR activation in the presence of costimulation (cross-linked anti-CD3 and anti-CD28 antibodies) for 5 min before cocultures were separated. HLA-A2^+^ cells were pre-labeled with anti-HLA-A2-FITC antibodies and magnetic anti-FITC microbeads to enable fast and chilled coculture separation after stimulation, maintaining signaling phosphorylations in the cells as much as possible. For comparison, we studied unstimulated Tcons (Trest) from the same donors and treated in the same way in parallel. Re-purified responder Tcons were then lysed, proteins were digested, and peptides labeled using stable isotope dimethyl labeling, before pooling, phosphopeptide enrichment by titanium ion immobilized metal affinity chromatography (Ti^4+^ IMAC), and LC-MS/MS (Figure 1A).

We introduced several quality control checkpoints. Purity of CD4^+^CD25^−^ Tcons and CD4^+^CD25^++^FOXP3^+^ Tregs was controlled by staining cells isolated from peripheral blood for expression of CD4, CD25, and FOXP3 (Figures S1A–C in Supplementary Material). We re-ensured a ~1:1 cell ratio in cocultures by analyzing the expression of HLA-A2 from aliquots of the respective cocultures (Figure S1D in Supplementary Material). We further verified a high purity of re-isolated HLA-A2^−^ responder Tcons that were used for phosphoproteomic analysis after coculture separation (Figure S1D in Supplementary Material). Importantly, we measured the inhibition of *IL2* and *IFNG* expression in aliquots of Tsup compared to Tstim after 3 h of TCR stimulation to reflect upon the efficiency of Treg-mediated suppression, confirming 40–86% suppression of cytokine mRNA in the cultures from the three phosphoproteomics donors (Figure 1B). Similar levels of these cytokine mRNAs in Tcons stimulated in allogeneic (Tstim) or autologous cultures as a further control suggested that the allogeneic setting did not substantially affect responder Tcons (Figure 1B). Finally, we verified a ~1:1:1 ratio of the differentially dimethyl-labeled peptides in the peptide pool by MS (data not shown).

### The Phosphoproteomic Landscape of Tcons Changes Specifically upon TCR Stimulation and Treg-Mediated Suppression

In the primary T cell samples, we detected 2,137 phosphopeptides corresponding to 786 proteins from the 3 donors used. 1,017 phosphopeptides (48%) were detected in at least 2 out of 3 donors (Figure 1C), and in further quantitative summary and statistical analyses we considered only those phosphopeptides. Given the limited primary sample material, it has to be considered that not all phosphoproteins are detectable with the MS approach used, and we assessed the coverage of known TCR signaling phosphoproteins detected by comparison with the Kyoto Encyclopedia of Genes and Genomes (KEGG) database (36). From ~60 signaling proteins (not all of which are regulated by phosphorylation upon TCR stimulation) reported to be involved in the TCR signaling pathway according to KEGG, we detected 15 phosphoproteins in our study of which 11 were detected in ≥2 out of 3 donors (Figure S2 and Table S1 in Supplementary Material; peptides detected in single donors were not quantified but can be found in the raw data in the repository).

**Figure 2:**
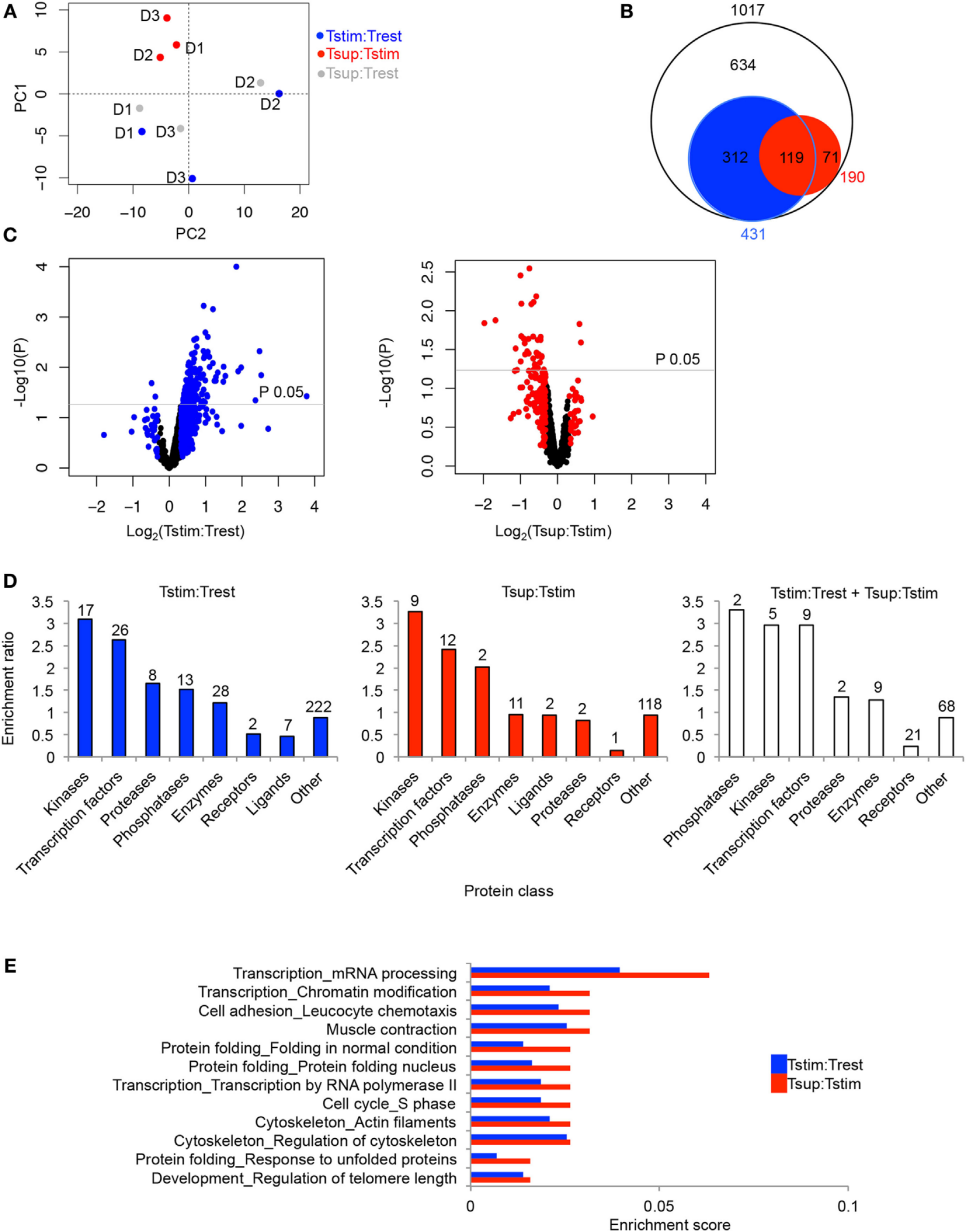
Activation- and Treg-induced changes in the phosphoproteome of Tcons. Unique phosphopeptides detected in ≥2 out of 3 donors were considered for summary analyses. The ratios reflecting changes upon TCR activation (Tstim:Trest), Treg-mediated suppression (Tsup:Tstim), and suppression compared to the unstimulated state (Tsup:Trest) are represented in blue, red, and gray, respectively. In addition, filtering was done in **(B–E)** to focus on the regulated phosphopeptides changing ≥25% in respective comparisons. **(A)** Principle component analysis (PCA) was performed based on the given phosphopeptide ratios for all three donors (D1, D2, D3). **(B)** Circles represent the total number (white), TCR stimulation-regulated (blue), and suppression-regulated (red) phosphopeptides along with the overlaps. Circle sizes are proportional to the numbers. **(C)** Volcano plots show stimulation-induced (left) and suppression-induced changes (right). The black dots represent unique phosphopeptides with <25% change. *P* values were calculated with Bayes moderated *t* test in limma package in R. log_2_ of average ratios of phosphopeptides are plotted against −log_10_ of *P* values in the particular comparison. **(D)** Enrichment ratio for respective classes of phosphoproteins regulated upon activation (blue), suppression (red), and jointly upon activation and suppression (white). Enrichment ratio was calculated as ratio between actual number of proteins (numbers above bars) and expected number of proteins enriched in individual categories according to “Enrichment by protein function” analysis in MetaCore. **(E)** Enrichment scores for process networks enriched (*P* < 0.05) upon Treg-mediated suppression are paired with corresponding process networks upon TCR stimulation and are arranged according to descending enrichment values in Tsup:Tstim. Enrichment score was calculated as ratio between number of items from the respective MetaCore process present in the lists and total items enlisted under the process. Total number of proteins detected in ≥2 out of 3 donors was set as background.

The effects of TCR stimulation and Treg-mediated suppression on the phosphoproteome of Tcons were relatively quantified by calculating the peptide intensity ratios between the three conditions (Trest, Tstim, and Tsup). Stimulation-induced changes are represented by the ratio Tstim:Trest and depicted in blue, while Treg suppression-induced changes on the stimulated state are reflected in the ratio Tsup:Tstim and presented in red (Figure 1A). Tsup:Trest represented in gray provides a comparison between the suppressed and resting state of Tcons. Principal component analysis (PCA) showed a donor-independent grouping based on suppression (Figure 2A), suggesting that Tregs enforced a unique signature on the phosphoproteome of Tcons.

Phosphoproteomics, particularly with scarce primary sample material, is generally limited in coverage and effect size as opposed to, for example, transcriptomics or even total proteomics. Therefore, we first considered those phosphopeptides which exhibited at least 25% change between two conditions, to gain a general picture of global changes without further statistical cutoffs. Regarding the proteins assigned to TCR signaling in the KEGG database (Figure S2 in Supplementary Material), almost all (10/11) of those quantified (i.e., detected in ≥2 out of 3 donors) also changed their phosphorylation status upon TCR stimulation, which was not affected by Tregs in most cases according to the applied 25% cutoff (Table S1 in Supplementary Material). Overall, we observed a massive alteration of the phosphoproteomic landscape upon TCR stimulation (compared to the resting state) as well as upon Treg-mediated suppression (compared to the stimulated state; Figures 2B,C; Table S1 in Supplementary Material). Out of 1,017 phosphopeptides, 431 phosphopeptides (42%) were changing upon TCR stimulation and 190 phosphopeptides (19%) were changing upon Treg-mediated suppression (Figure 2B). Out of these 190 phosphopeptides changing upon suppression, 119 (63%) were also changing upon stimulation in the absence of Tregs, whereas 71 (37%) were changing exclusively upon suppression with the 25% change cutoff applied.

Next, we examined the directionality of the changes. Out of all phosphopeptides changing upon TCR stimulation (431 peptides in total), 92% exhibited elevated phosphorylation (Figure 2C). In stark contrast, Tregs seemed to reverse the stimulation-induced phosphoproteomic changes with the majority of phosphopeptides (158 phosphopeptides, 83% of 190 phosphopeptides changing upon suppression) exhibiting reduced phosphorylation upon Treg-mediated suppression (Figure 2C). Out of these 158 phosphopeptides, 105 phosphopeptides at the same time exhibited higher phosphorylation upon stimulation (Table S1 in Supplementary Material), suggesting that Tregs prevent stimulation-induced phosphorylation of these peptides. Notably, these 105 peptides changing in opposite direction between stimulation and suppression represented the majority (88%) of those 119 peptides that changed in both comparisons (Figure 2B; Table S1 in Supplementary Material). The remaining 53 phosphopeptides with Treg-induced dephosphorylation seemed to be not simply the result of the inhibition of TCR stimulation-induced phosphorylation, but included phosphorylations exclusively changing upon Treg-mediated suppression and not appearing changed upon TCR stimulation by itself.

These results are the first indication that Tregs globally regulate the phosphoproteome of Tcons, and they suggest that Tregs suppress Tcons by maintaining or enhancing a resting state similar to unstimulated Tcons as well as by inducing novel changes unrelated to TCR stimulation.

### Global Analysis of Processes Related to Phosphorylation Events Changing upon TCR Stimulation and Treg-Mediated Suppression

Although it needs to be noted that the 25% change cutoff does not necessarily coincide with statistically significant changes considering donor variation, we restricted this first global part of our analysis to this cutoff to obtain large enough numbers of elements in protein lists to study general changes in cellular pathways. To this end, we analyzed whether the global phosphoproteomic changes occurring in Tcons upon TCR stimulation and Treg-mediated suppression were enriched for involvement of certain pathways and types of proteins. The dominant protein classes appearing among phosphoproteins changing upon stimulation or suppression (Figure 2D, blue and red bars, respectively) were transcription factors and enzymes, specifically kinases, proteases, and phosphatases. Interestingly, phosphatases, kinases, and transcription factors were most enriched among phosphoproteins that changed upon both stimulation and suppression (Figure 2D, white bars). In addition, we performed comparative gene ontology (GO) analysis to examine whether process networks enriched upon suppression were also enriched upon stimulation without Tregs (Table S2 in Supplementary Material). Almost all processes significantly enriched (*P* < 0.05) upon suppression were also significantly enriched upon stimulation alone (Table S2 in Supplementary Material). TCR stimulation revealed additional significantly enriched processes not encompassed upon suppression, presumably due to the higher number of proteins changing upon stimulation as compared to suppression (Figure 2B; Table S2 in Supplementary Material). Considering specifically the processes enriched (*P* < 0.05) in suppressed cells, these included protein folding, various transcription-related processes, cell cycle, cell adhesion involved in leukocyte chemotaxis, and various cytoskeletal regulation processes, among others (Figure 2E; Table S2 in Supplementary Material). Transcriptional and translational regulation as well as proliferation are integral parts of T cell stimulation which are a potential target for Treg-mediated suppression. Furthermore, processes affecting cytoskeletal regulation have been shown in multiple instances to mediate early events of TCR signaling including Ca^2+^ and NFAT signaling in T cells (17, 18, 22) the latter being a causative mechanism for Treg-mediated suppression of cytokine transcription (24).

### Tregs Counter Stimulation-Induced Phosphorylation in Responder Tcons

Before further detailed analysis of specific suppression-induced changes, we applied a more stringent cutoff than the 25% change, considering only phosphopeptides changing in a statistically significant manner. Considering the subset of the phosphopeptides that changed significantly (*P* < 0.05) in any of the three comparisons (i.e., ratio Tstim:Trest, Tsup:Tstim, and Tsup:Trest) and comparing the individual ratios over all the donors, a donor-independent clustering based on suppression was observed (Figure S3 in Supplementary Material). Together with the above results from the PCA analysis on all the data without filtering (Figure 2A), these results suggest that the suppression-induced effect was overriding the donor variability. Furthermore, it was confirmed with this statistically stringent cutoff that the majority of proteins that displayed an increased phosphorylation upon stimulation at the same time exhibited reduced phosphorylation upon suppression (Figure S3 in Supplementary Material), similar to the above analysis without *P* value cutoff (Figure 2C).

**Figure 3:**
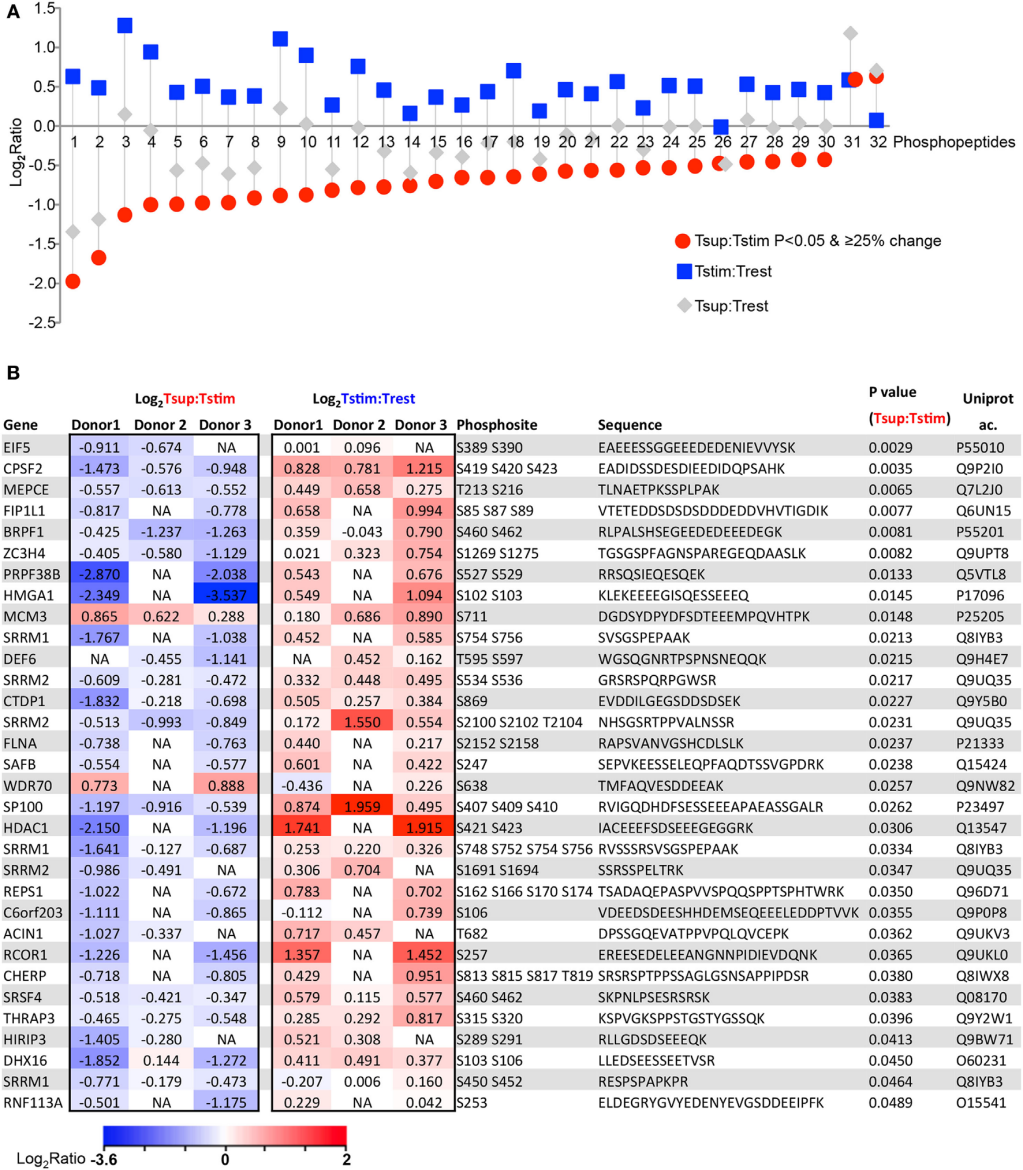
Tregs counter activation-induced phosphorylation in responder Tcons. 32 phosphopeptides (corresponding to 28 proteins) change significantly (increased: 2, decreased: 30) upon Treg-mediated suppression (*P* < 0.05 and changing ≥25% in Tsup:Tstim). **(A)** Changes are represented as log_2_ of average ratios for the indicated comparisons. Peptides are arranged by increasing log_2_ ratio in Tsup:Tstim. **(B)** The 32 phosphopeptides with respective phosphosites and corresponding sequences and gene names are presented as individual log_2_ ratio in 3 donors upon suppression and stimulation. Heat map color scale represents log_2_ fold change ratio values.

Next, we focused on the Treg suppression-induced changes more specifically, with the aim to define novel molecules that cause Treg-mediated suppression of Tcon cytokine expression. To choose a relevant “candidate protein” for functional verification, we applied a most stringent combined cutoff on the candidate protein list: we focused only on the subset of phosphopeptides that changed significantly (*P* < 0.05) upon suppression, and that was also changed at least 25% in Tsup versus Tstim (here defined as “significantly changing upon suppression”). 32 phosphopeptides (corresponding to 28 proteins) were significantly changing upon Treg-mediated suppression (Figure 3). Almost all phosphopeptides that were significantly dephosphorylated upon suppression were found to have a tendency of being phosphorylated upon TCR stimulation (29 out of 30), suggesting that Tregs prevent the TCR stimulation-induced phosphorylation (Figures 3A,B) and confirming the indications from above studies without statistical cutoff (Figures 2B,C). For several of these phosphoproteins, Tregs did not only hinder stimulation-induced phosphorylation but even pressed the phosphorylation status below that of unstimulated Tcons, at least when considering the mean change averaging donors without statistical cutoffs (Trest; gray symbols in Figure 3A).

Together, these results demonstrate that Tregs impose a unique profile on the phosphoproteome of Tcons mainly by countering the stimulation-induced phosphorylation. Notably, many of the shortlisted phosphoproteins discovered in our unbiased study are neither known to be involved in TCR signaling nor in Treg-mediated suppression. Hence, this list of phosphoproteins may be useful to unravel novel mediators of Treg-mediated suppression and T cell activation in this context.

### Tregs Suppress Phosphorylation of the Actin Cytoskeleton- and Ca^2+^/NFAT-Regulator DEF6 in Responder Tcons

Since we were studying the phosphoproteomic changes within 85 min of coculture and shortly after TCR stimulation (5 min), we suspected that some of the shortlisted phosphorylation changes may represent causative and proximal events mediating the unknown mechanism of Treg-induced rapid suppression of signaling and cytokine expression in Tcons. We performed GO analysis of the 32 shortlisted phosphopeptides, which indicated enrichment of pathways regulating cytoskeletal elements (Table S3 in Supplementary Material), although it needs to be noted that the number of query proteins was limited and hence the results are indications only. Yet, processes involved in cytoskeletal regulation were accordingly also enriched upon suppression in the GO analysis using the extended list of phosphoproteins (25% change cutoff; Figure 2E). Together, these observations support the notion that the proximal events for Treg-mediated rapid suppression of Tcons may be shaped by phosphoproteins involved in cytoskeletal regulation.

The role of cytoskeletal rearrangement in T cell signaling is not fully understood. Out of several molecules involved in cytoskeletal regulation in our candidate protein list, DEF6 also known as SLAT, has been shown to regulate Ca^2+^/NFAT signaling in CD4^+^ T cells by interaction with the IP_3_R (23) and activation of cytoskeleton-regulating Rho-family GTPases (22, 37). Despite its known role in cytoskeleton regulation and TCR signaling, DEF6 is not represented in the relevant processes in commonly used databases such as KEGG, MetaCore, and String yet. DEF6 possesses a non-canonical catalytic Dbl homology (DH) domain that has been shown to be crucial to exert its guanine nucleotide exchange factor activity toward Rho-family GTPases. Following TCR stimulation, DEF6 translocates to the immunological synapse and is involved in the TCR stimulation-induced activation of NFAT and T cell cytokines. Interestingly, a membrane-targeted DEF6 DH domain alone was necessary and sufficient for these effects (22). Despite its crucial role in DEF6 activity, the specific residues in the DH domain responsible for DEF6 activity are largely unknown, possibly due to low primary amino acid sequence homology of the DEF6 DH domain with canonical DH domains.

**Figure 4:**
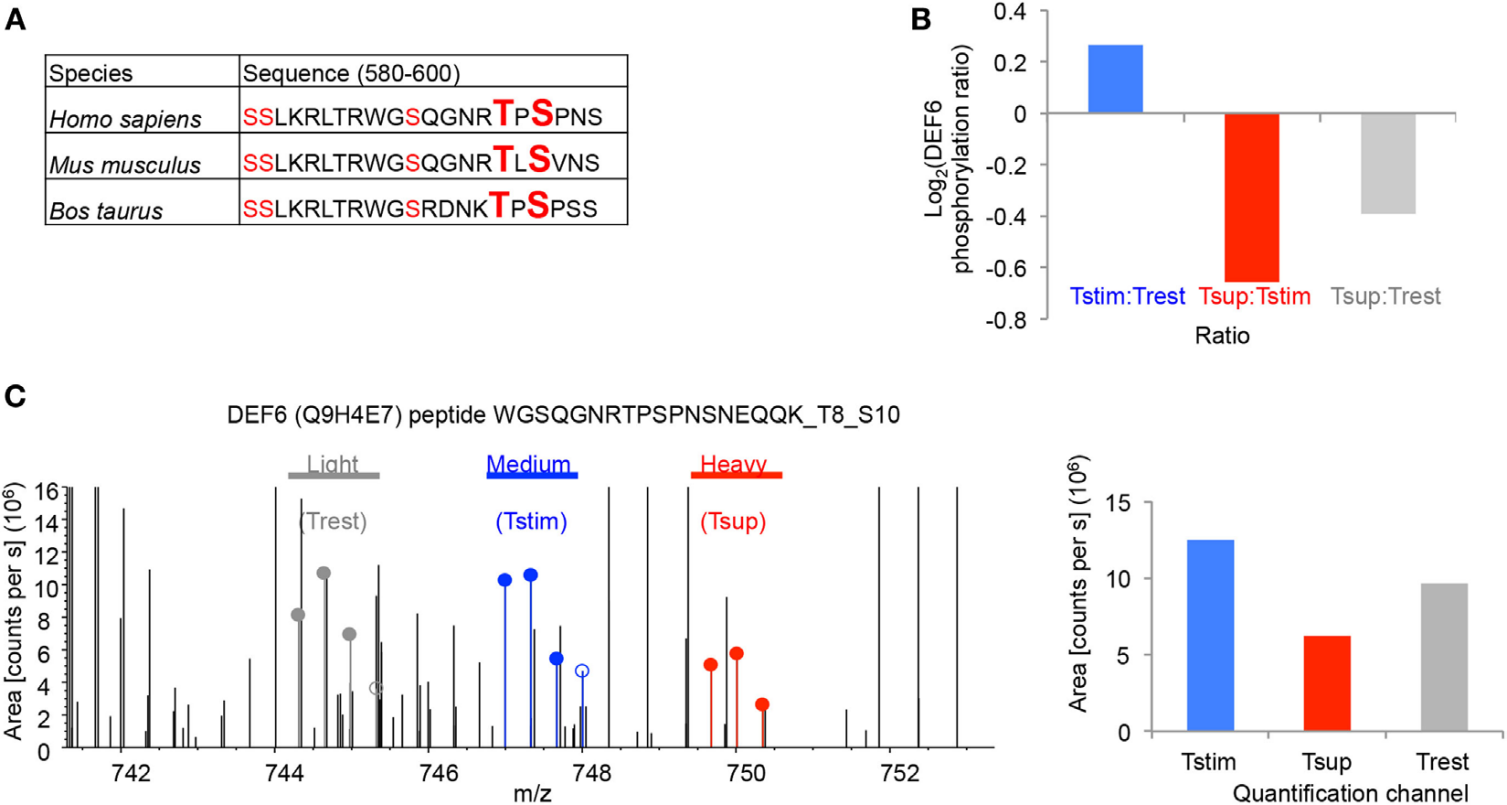
Tregs suppress T595_S597 phosphorylation of DEF6 in responder Tcons. **(A)** The peptide sequences of DEF6 containing the conserved phosphosites in respective organisms are shown. Ser (S) and Thr (T) with detected phosphorylation in ≥2/3 donors are highlighted in red. Phosphosites of interest (T595 and S597) are additionally highlighted in big letters. **(B)** log_2_ of average ratio of T595_S597 phosphorylated DEF6 peptide in the given comparisons. **(C)** Representative FTMS spectrum of the indicated DEF6 phosphopeptide (phospho-T595_S597). Precursor areas of the three indicated samples are depicted. Shown is the spectrum from Donor 3 (from one out of three technical replicate mass spectrometry runs) representative of three donors, with following properties: quantified Ion: *z* = +3, Mono *m*/*z* = 744.31616 Da, and MH+ = 2,230.93393 Da. Filled circles are isotope pattern peaks used in calculating the quantification values for the different quantification channels, as opposed to unfilled circles. The bar chart (right) shows quantification of the respective spectrum.

We detected a total of five phosphosites in DEF6 all of which were mapped to the DH domain but are not functionally characterized (Figure 4A). These phosphosites were conserved in mammals including mice and men (Figure 4A). Importantly, our data show that Tregs significantly reduced phosphorylation of DEF6 T595 and S597 compared to Tcons activated in the absence of Tregs (Figures 3B and 4B,C). Furthermore, TCR stimulation enhanced the phosphorylation of DEF6 T595 and S597 compared to unstimulated cells in both donors in which the respective peptide was detected, albeit the activation-induced phosphorylation did not pass the abovementioned cutoffs of 25% average change or *P* < 0.05 (Figures 3B and 4B,C; Table S1 in Supplementary Material). DEF6 T595 and S597 phosphorylation seemed reduced upon Treg-mediated suppression even below the unstimulated state, although this trend was not statistically significant (Figures 4B,C; Table S1 in Supplementary Material). Suppression-induced reversal of stimulation-induced upregulation of DEF6 T595_S597 phosphorylation was observed in multiple (although not all) phosphopeptides harboring the particular phosphosites in individual donors (Figure S4 in Supplementary Material). Within the quantified phosphoproteomics data, the respective DEF6 T595_S597 phosphopeptide was detected only in 2 out of 3 donors and hence to further support the results, we studied an additional verification donor. Notably, we confirmed the pattern of DEF6 T595_S597 phosphorylation upon TCR stimulation and Treg-mediated suppression in the additional independent verification donor who was omitted from the above summary and statistical analysis due to overall lower coverage of detected phosphopeptides (Figure S4 in Supplementary Material). Since the DH domain is crucial for DEF6 activity, we hypothesized that T595_S597 phosphorylation in the DH domain may be involved in controlling the functionality of DEF6 in T cell activation and Treg-mediated suppression.

### T595 and S597 Phosphosites in the DEF6 Protein Are Crucial for Its Interaction with the IP_3_R

**Figure 5:**
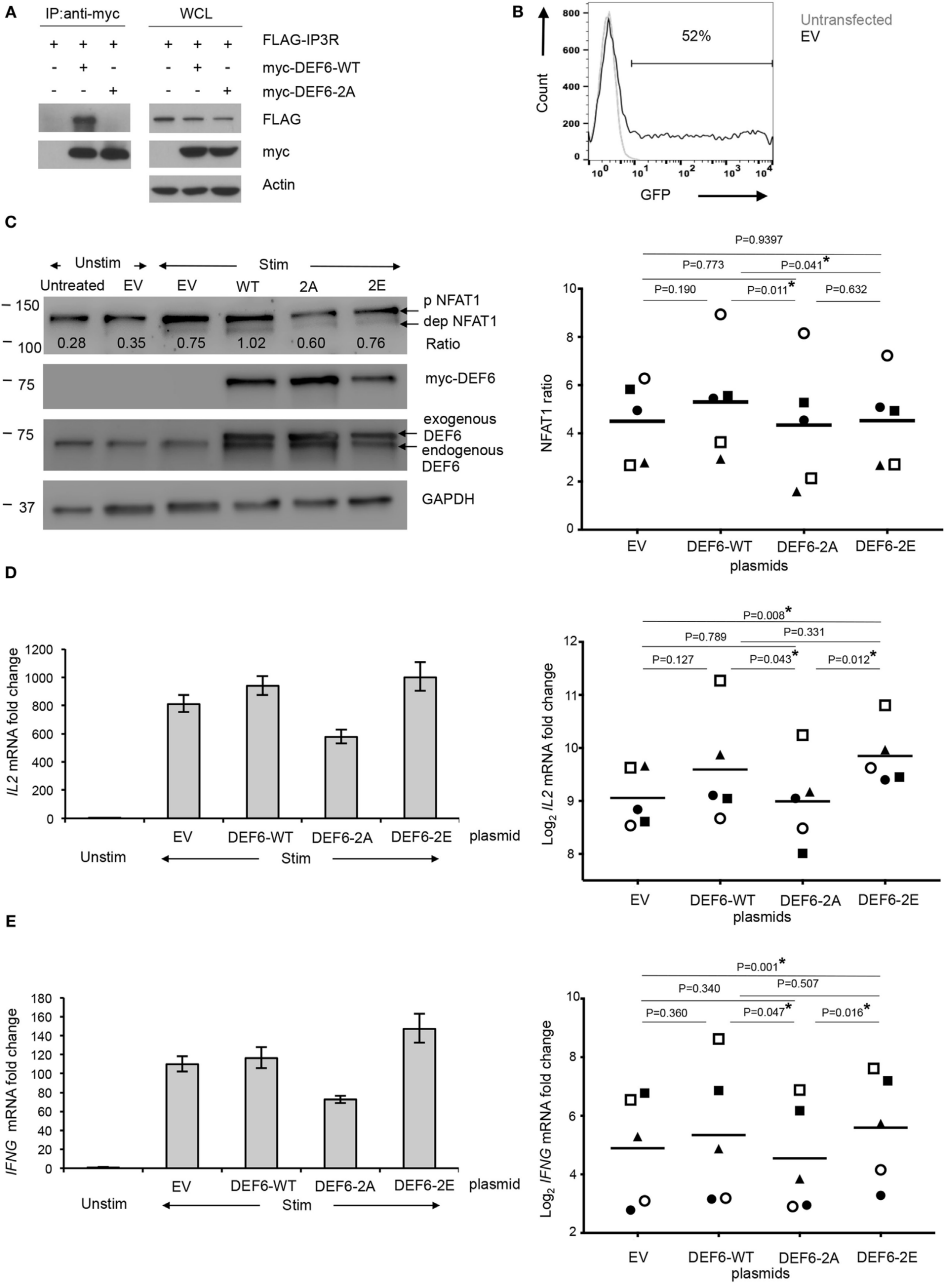
T595 and S597 phosphosites in DEF6 protein contribute to IP_3_R interaction and T cell activation. Plasmids encoding for DEF6-WT, DEF6 T595_S597 phospho-mutant (DEF6-2A), and DEF6 T595_S597 phospho-mimic (DEF6-2E) were generated. **(A)** HEK293 cells were cotransfected with indicated myc-tagged DEF6 constructs along with FLAG-tagged IP_3_R. Whole cell lysates (WCLs) were subjected to immunoprecipitation (IP) with anti-myc antibody (left), and aliquots of WCLs were kept untreated (right). Western blot antibodies are indicated. A representative experiment of 2 is shown. **(B–E)** Primary human Tcons were transiently transfected with plasmids encoding the indicated DEF6 protein or empty vector [EV; green fluorescent protein (GFP) in place of DEF6] for 8 h. Where indicated, cells were subsequently stimulated (Stim) with anti-CD3/anti-CD28 antibodies and processed as follows: **(B)** transfection efficiency based on GFP expression in EV-transfected cells was determined by flow cytometry. It was pre-gated on live lymphocytes based on fsc/ssc. Overlaid histogram of GFP expression in untransfected and EV-transfected Tcons is representative of five donors in two independent experiments. **(C)** Cells were stimulated for 5 min and analyzed by Western blot with antibodies against indicated targets (left). The ratio of levels of dephosphorylated NFAT1 and phosphorylated NFAT1 was calculated after quantification of the bands. Expression levels of myc-tagged DEF6 or total DEF6 and GAPDH served as transfection and loading controls, respectively. Blot is representative of five donors. Right: NFAT1 ratios were normalized to the corresponding NFAT1 ratio in unstimulated Tcons of the same donor which was set to 1. Individual NFAT1 ratios from *n* = 5 donors (represented by individual symbol per donor) from 2 independent experiments along with the mean values (line) are shown. *IL2* **(D)** and *IFNG* **(E)** mRNA in transfected Tcons after 3 h of stimulation or without stimulation (Unstim), normalized to *RPL13A* mRNA. Results are presented as fold change compared to unstimulated Tcons of the same donor, which was set to 1. Left: mean ± SD of technical triplicates from quantitative RT-PCR is shown for one donor, representative of five donors from two independent experiments. Right: individual values of log_2_ fold change of mRNA levels of the respective cytokines from five donors (represented by individual symbol) are shown along with mean values (lines). *P* values **(C–E)** were determined by paired, two-sided Student’s *t*-test (*P < 0.05).

To investigate the functional relevance of T595_S597 phosphorylation of DEF6, we created plasmids for expression of wild-type human DEF6 (DEF6-WT) or a phospho-silent DEF6 mutant in which both T595 and S597 were substituted by alanine (DEF6-2A). Because we previously determined that direct interaction of DEF6 with the IP_3_R regulates the Ca^2+^/NFAT-mediated activation of T cells (23), and that Tregs inhibit Ca^2+^/NFAT signaling in Tcons downstream of TCR-proximal signaling including IP_3_ generation yet suppressing Ca^2+^ store depletion (24), we hypothesized that T595_S597 phosphorylation of DEF6 may contribute to the interaction with the IP_3_R and, thus, T cell activation. To study the involvement of DEF6 T595_S597 phosphorylation in DEF6:IP_3_R interaction, we performed coimmunoprecipitation assays of myc-tagged DEF6 variants and IP_3_R using HEK293 cells. While DEF6-WT bound to the IP_3_R, this DEF6:IP_3_R interaction was obliterated upon mutation of the T595_S597 phosphosites (Figure 5A). These results indicate a novel role of the phosphorylated T595_S597 residues in the DEF6 DH domain, crucial for DEF6 interaction with the IP_3_R.

### DEF6 Protein T595_S597 Phosphorylation Contributes to T Cell Activation and Cytokine Expression

Next, we strived to study the functional relevance of our findings in primary human Tcons. However, overexpression of plasmids in unstimulated primary human T cells without seriously affecting cellular viability and stimulatability poses a serious challenge. While overexpression in primary T cells can be achieved with lentiviral systems, efficient transduction usually requires pre-activation (38, 39) and/or affects stimulatability of the cells (40) and, thus, is not suitable for the study of early cytokine mRNA expression and signaling immediately after TCR stimulation. To the best of our knowledge, only few reports have successfully transfected constructs for ectopic protein expression into primary T cells, usually utilizing the Amaxa nucleofection technique (41–43). However, this technique results in relatively low cell viability particularly with increasing DNA amounts and transfection efficiency, and the procedure was also shown to affect T cell stimulation including calcium signaling (44). We also observed low viability and compromised activation of Tcons after Amaxa transfection of plasmids (data not shown); hence, we tested transient transfection of plasmids in T cells with the Neon transfection system that offers versatility allowing electroporation parameters to be optimized freely. After optimizing transfection parameters, doses and incubation times using the EV corresponding to the DEF6 expression plasmids above but with green fluorescent protein (GFP) in place of DEF6, we attained 48–52% transfection efficiency as assessed by GFP expression (Figure 5B) while retaining high viability and relatively high capacity to respond to TCR stimulation. To investigate whether T595_S597 phosphorylation of DEF6 plays a functional role in NFAT activation, we compared the levels of activated NFAT in lysates from Tcons transfected with plasmids encoding for DEF6-WT, DEF6-2A, or EV after 5 min of TCR stimulation (Figure 5C). With the optimized and titrated plasmid amounts used, ectopic expression of DEF6 did not result in artificially high DEF6 amounts on the cell population level, but we attained comparable levels of exogenous DEF6 in reference to endogenous DEF6 expression (Figure 5C). Accordingly, DEF6 overexpression did not drastically increase NFAT activation compared to EV transfection (Figure 5C) despite a trend in agreement with the known positive effect of DEF6 on T cell stimulation (22, 45). Importantly, we observed that expression of DEF6-2A significantly lowered the activation of NFAT as compared to expression of DEF6-WT in Tcons from all five donors tested (*P* < 0.05; Figure 5C). We also tested the reverse approach using a DEF6 phospho-mimic mutant in which replacement of the T595_S597 sites with glutamic acid (DEF6-2E) should mimic the negative charge of phosphorylation. However, overexpressing this phospho-mimic mutant did not lead to an increase in rapid NFAT activation (Figure 5C).

In Tcons, the direct effect of NFAT activation (together with T cell activation-induced transcriptional cofactors) is reflected in transcription of activation-induced genes such as *IL2* and *IFNG*. Our finding from the studies with the DEF6-2A phospho-silent mutant that T595_S597 phosphorylation of DEF6 was involved in NFAT activation would have functional relevance if it would also affect the expression of such T cell activation-induced genes. Indeed, qRT-PCR analysis of *IL2* and *IFNG* mRNA revealed that Tcons transfected with DEF6-2A expressed significantly lower amounts of both *IL2* and *IFNG* mRNA compared to DEF6-WT-transfected Tcons upon 3 h of TCR stimulation in all five donors tested (Figures 5D,E). Furthermore, DEF6-2A mutation also resulted in significantly lower cytokine expression compared to the phospho-mimic DEF6-2E mutant in all five donors tested (Figures 5D,E). Transfection of the DEF6-2E mutant led to a significant increase in cytokine expression compared to EV transfection, as well as to a mild (in four of five donors) albeit not significant upregulation of cytokine mRNA compared to DEF6-WT transfection (Figures 5D,E). We also observed the same trend of reduced activation upon DEF6-2A overexpression versus DEF6-WT or versus DEF6-2E for other T cell activation-induced NFAT target genes *viz*. *IL2RA* (encoding for CD25) and *FASLG* (Figure S5 in Supplementary Material). Hence, our results indicate that these DEF6 phosphosites positively regulate T cell activation upon phosphorylation.

**Figure 6:**
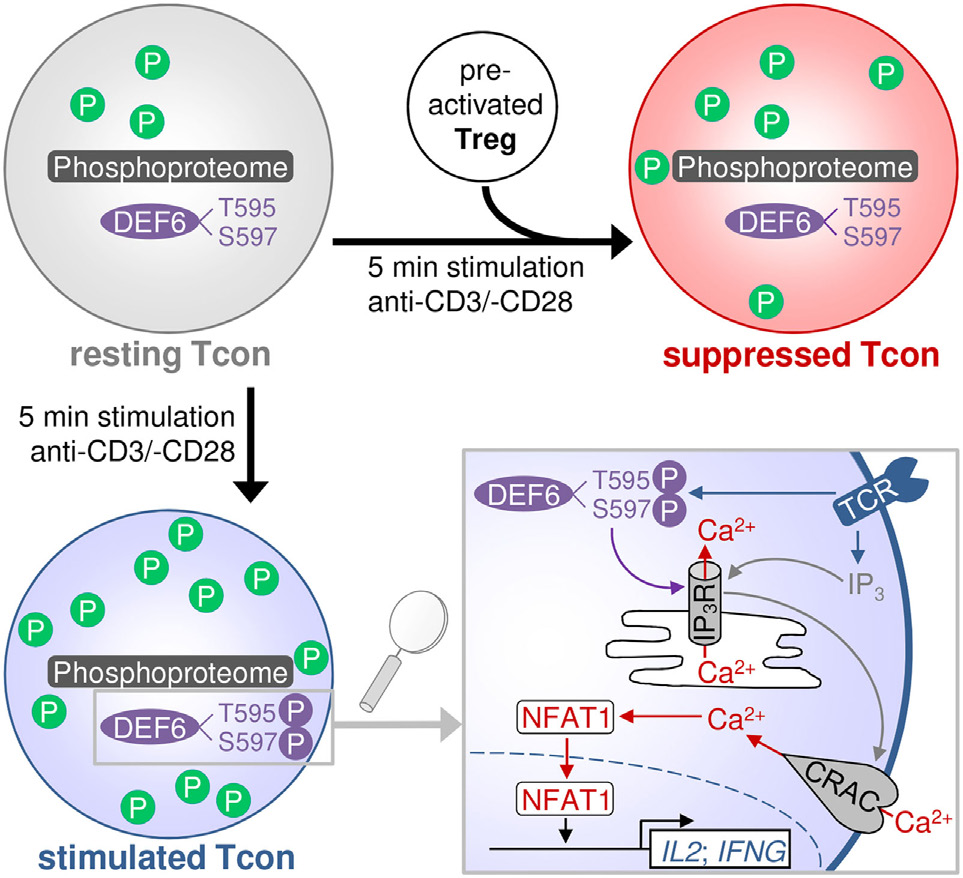
Proposed model of activation- and suppression-induced changes in the Tcon phosphoproteome. The scheme depicts the general upregulation of phosphorylation (P) upon TCR stimulation, which is largely counteracted by Tregs. Treg-suppressed cells partially resemble the unstimulated state and also present some additional changes not occurring upon activation. Tregs inhibit DEF6 phosphorylation at T595 and S597 sites, which is proposed to occur upon TCR activation and to confer interaction with the IP_3_R and downstream NFAT signaling and cytokine expression. Lower right panel modified from Ref. (8).

Altogether, we here provide a resource of quantitative phosphoproteomic data of primary human Tcons in the resting, TCR-stimulated, and Treg-suppressed TCR-stimulated states. Our results demonstrate a global change in phosphorylation upon TCR stimulation that is predominated by upregulation of phosphorylation while Tregs induce a state dominated by dephosphorylations. Tregs counter the stimulation-induced phosphorylation state as well as change the phosphorylation status of proteins that were not affected by stimulation (Figure 6). We shortlisted several phosphoproteins having a putative role in Treg-mediated rapid suppression of Tcons. We newly identified the phosphosites T595 and S597 in DEF6. These sites showed a trend of increased phosphorylation upon TCR stimulation and conversely were significantly dephosphorylated upon Treg-mediated suppression. Our functional validation results demonstrate a role of these phosphosites in DEF6 interaction with the IP_3_R, as well as in NFAT activation and subsequently transcription of activation-induced genes in primary Tcons. We present insights into a novel proposed molecular mechanism by which T595_S597 phosphorylation of DEF6 promotes cytokine expression in Tcons and which is a target of Treg-mediated suppression (Figure 6).

## Discussion

We provide a resource of phosphoproteomic data from primary human CD4^+^CD25^−^ T cells in the resting, TCR-activated, and Treg-suppressed state, including functional validation of novel phosphorylations regulated upon TCR stimulation and suppression. We observed a globally enhanced phosphorylation state upon TCR stimulation, and conversely an overall decrease of phosphorylation in Treg-suppressed cells compared to TCR stimulation alone. Although an overall increase in phosphorylation upon TCR stimulation is expected, only few studies have measured the phosphoproteome in primary human T cells on a global scale like in the resource we provide. Most existing studies of the T cell phosphoproteome are limited to the Jurkat T cell line or primary murine T cells. Unbiased studies on the phosphoproteome of human primary cells upon TCR stimulation have been performed with bulk lymphocytes containing ~80% CD3^+^ T cells (46, 47), while a recent report using highly purified human naïve and memory CD4^+^ T cells only studied unstimulated cells (48). In accordance with our results in human primary CD4^+^CD25^−^ T cells, a paramount increase in protein phosphorylation upon TCR stimulation has also been reported in phosphoproteomic studies of murine primary CD4^+^ and CD8^+^ T cells (49–51).

The majority of well-studied TCR-induced phosphorylations are tyrosine (Tyr, Y) phosphorylations while Ser/Thr phosphorylations are less well studied although their TCR-induced changes outnumber Tyr phosphorylation events (47, 52). One reason may be that numerous previous studies have focused on Tyr phosphorylation events using biased pre-enrichment of these through immunoprecipitation. We here employed a Ti^4+^ IMAC phosphopeptide enrichment method that was demonstrated to have high specificity and to lack apparent biases regarding biochemical and biophysical peptide parameters including the type of phosphorylated amino acid (31, 53). Thus, the obtained phospho amino acid content ratio reflects the naturally occurring ratio in vertebrate cells (pSer:pThr:pTyr = 1,800:200:1) (54, 55). Due to their naturally rare occurrence, pTyr residues are therefore poorly covered in such an unbiased approach. However, the low amount of obtainable input material using highly purified subsets of human primary T cells as in our study did not allow us to perform a parallel enrichment of Tyr and Ser/Thr phosphorylations. A study in the Jurkat T cell line that combined phosphopeptide enrichment by strong cation exchange (SCX) fractionation or by immunoprecipitation of pTyr-containing peptides, and IMAC, identified ~700 TCR-responsive phosphorylation sites, of which ~10% were pTyr residues (52). In a study of primary human lymphocytes with massive amounts of input material (30 mg protein) allowing for both pTyr immunoprecipitation and phosphopeptide enrichment with TiO_2_, ~1.7% of detected phosphopeptides were phosphorylated on Tyr (47). Despite a much more purified hence more homogeneous cell population and ~200-fold less protein input in our study compared to the mentioned study by Ruperez et al. (47), the number of unique phosphopeptides we detected was still more than 3/4th of the number these authors detected (2,137 phosphopeptides from 786 proteins versus 2,814 phosphopeptides from 1,372 proteins); both studies included unstimulated and 5 min TCR-stimulated cells although the number of donors differs between the studies. Thus, our data will be a valuable complement to previous studies such as those in the LymPHOS database that currently contains ~4,000 confidently quantified phosphosites of human lymphocytes with a Ser:Thr:Tyr ratio of ~100:10:1 (46). Notably, even with parallel enrichment of pTyr and pSer/pThr in one of these studies, the majority of phosphorylations changing upon TCR activation in human lymphocytes were Ser/Thr phosphorylations (47).

Given the importance of CD4^+^ T cells in the human immune system, our unique phosphoproteome data from primary human CD4^+^ T cells are highly relevant from the perspective of immunotherapy. Even more importantly, studies on the phosphoproteome in Treg-suppressed T cells are completely absent to date and hence, our resource is valuable particularly in light of autoimmune diseases. Well-known TCR signaling proteins and second messengers have been previously studied in suppressed T cells (24, 26, 27), but only in a targeted manner restricted to known phosphosites without revealing the exact mechanism of suppression. It is worth noting that those proteins we revealed here as differentially phosphorylated upon suppression and for which Tregs prevented activation-induced phosphorylation were not the “classically” known TCR signaling pathway proteins, but included many proteins previously unknown in this context. This highlights the importance of an unbiased approach and is in line with previous findings showing that Tregs do not generally inhibit all the classical TCR signaling pathways in responder T cells (24, 27).

It should be noted that although this study is focused on phosphorylation, other post-transcriptional events and PTMs that regulate TCR signaling may be involved in Treg-mediated suppression as well. An important example is the emerging complex modulation of TCR signaling by ubiquitination and ubiquitin-like modifications such as SUMOylation and neddylation (56). Another example is arginine methylation that modulates TCR and CD28 signaling (57). A TCR pathway heavily regulated by PTMs is the NF-κB pathway, which involves not only phosphorylation but also protein acetylation, methylation, and ubiquitination including linear ubiquitination (58, 59). Such modifications involved in TCR signaling may confer rapid or long-term Treg-mediated suppression as well, and future studies should address this topic. Recent advancements in MS-based approaches to measure such PTMs on a global scale (60, 61) provide the opportunity to study these modifications in a similar experimental setup as in our phosphoproteomic study in Treg-suppressed T cells.

In addition to the emerging role of PTMs in Treg suppression, regulation of expression levels of certain genes is a well-characterized phenomenon of Treg-mediated suppression especially over longer periods of time (8). Several of the phosphoproteins shortlisted in this study may have a novel role in suppression, potentially also in later mechanisms affecting transcription, protein folding, and cell cycle regulation as suggested by the GO analyses. High enrichment of phosphorylation events associated with transcriptional regulation likely represents initial phases of such regulation that will take effect at later time points, and which could be interesting for future studies on additional inhibitory mechanism that enhance and stabilize the early effects of Tregs also at later time points of T cell activation and suppression. In contrast, the rapid nature of Treg-mediated suppression of Tcon signaling and cytokine expression that we are studying here suggests that it is unlikely for transcriptional machineries to play a causative role in this aspect of suppression. Among the several pathways enriched upon suppression, cytoskeletal rearrangement, which has been shown in multiple instances to mediate early events of TCR signaling, was most relevant to the system under study. More importantly, it has been specifically shown to regulate Ca^2+^ and NFAT signaling in T cells (17, 18), and notably we have established Ca^2+^ inhibition as causative mechanism for rapid Treg-mediated suppression (8, 24). Hence, the phosphoproteins involved in cytoskeletal regulation appeared as interesting novel candidates to potentially mediate the rapid Treg-induced suppression.

Consequently, among our list of phosphoproteins significantly regulated by Tregs, we chose to follow-up the DEF6 protein because of its role in regulation of the cytoskeleton and rapid NFAT signaling in T cells (16, 22, 45, 62). We discovered novel functional Ser/Thr phosphosites in the DH domain of DEF6 and determined these to be phosphorylated upon TCR stimulation in three different donors, albeit not reaching statistical significance possibly due to high variation between donors in the absolute extent of stimulation-induced phosphorylation. Importantly, these sites were significantly dephosphorylated upon Treg-mediated suppression. Although these sites have been described in different cancer cell lines (63), S595 and T597 phosphosites were not previously quantified in human T cells even with unbiased phosphoproteomic studies (46, 47, 52) to our knowledge. So far, known TCR-regulated phosphosites of DEF6 have been restricted to Tyr phosphorylations in the immunoreceptor tyrosine-based activation motif (ITAM)-like sequence and mediating membrane recruitment of DEF6 (22, 37). The phosphosites we describe here locate to the DH domain of DEF6, and we observed complete abrogation of DEF6 interaction with the IP_3_R upon DEF6-2A mutation. However, Fos et al. observed that the DH domain alone did not associate directly with the IP_3_R while both EF-hand and PH domains of DEF6 directly interacted with the IP_3_R1 (23). Interestingly, upon enforced membrane-targeting, the DEF6 DH domain alone restored NFAT activation and IFN-γ expression in DEF6^−/−^ primary murine T cells to wild-type levels despite lack of the IP_3_R-interacting PH and EF-hand domains, suggesting an essential role of the DH domain (22). These results suggest that under proper localization to the membrane, the DH domain may mediate interaction of DEF6 with the IP_3_R and hence stimulate Ca^2+^/NFAT. In accordance with this notion, although EF-hand and PH domains were shown to independently and directly interact with the IP_3_R, deletion of both domains together (which leaves the ITAM-like motif and DH domain) still allowed for residual IP_3_R interaction and supported ~50% of NFAT activity (23), which may be attributed to the DH domain. Despite reducing IP_3_R interaction, neither deletion of the EF nor PH domain alone or together abrogated IP_3_R interaction completely (23), in contrast to the complete abrogation that we observed upon DEF6-2A mutation in the DH domain. Thus, in addition to affecting direct interaction of the DH domain with the IP_3_R as discussed earlier, mutation of the T595_S597 phosphosites may have structural effects on the DEF6 protein, hence explaining the complete loss of IP_3_R interaction in DEF6-2A mutants despite intact PH and EF-hand domains. Accordingly, the importance of conformational changes in TCR-induced DEF6 activation to allow for EF-hand and PH domain accessibility to the IP_3_R has been suggested before in the context of the ITAM-like DEF6 motif (23). Hence, it is plausible that upon TCR stimulation, phosphorylation of the DH domain indirectly modulates the DEF6:IP_3_R interaction by inducing conformational changes that affect other domains of DEF6 as well. How exactly the DEF6:IP_3_R interaction leads to increased Ca^2+^ flux is unclear to date, and two scenarios have been suggested: DEF6 serving as a Ca^2+^ sensor promoting Ca^2+^ binding to the IP_3_R, or DEF6 competing with IP_3_R-inhibiting other Ca^2+^-binding proteins (23). Although we validated a functional role using the phospho-silent DEF6 mutant (DEF6-2A), the reverse approach with “mimicking” the negative charge of phosphorylation by replacing the DEF6 T595_S597 phosphosites with glutamic acid led to more diverse results. Ectopic expression of this phospho-mimic mutant led to significant increase in cytokine expression over DEF6-2A mutant or EV expression, but only to mild increase over DEF6-WT. This mild difference suggests that negative charge of these phosphosites alone does not render DEF6 hyperactive. Altogether, our results suggest that in addition to the mere phosphorylation charge and previously assigned catalytic functions of the DEF6 DH domain, the phosphosites we discovered may have a role in conferring a DEF6 structure that allows for interaction with the IP_3_R through several domains including the DH domain itself, and consequently NFAT activation. Furthermore, this DEF6 DH domain phosphorylation was disturbed in Treg-suppressed T cells.

It should be discussed that the phospho-silent mutation led to almost complete abrogation of DEF6:IP_3_R interaction in HEK293 cell lines while the effect on cellular activation in primary human T cells was not as drastic, as measured by NFAT activation (average decrease 24%) and cytokine expression (average decrease 31% for *IL2* and 38% for *IFNG*). This might be attributed to the fact that T cells are more difficult to transfect and have higher expression of endogenous DEF6 as compared to HEK293 cell lines. In fact, transfected T cells expressed relatively low levels of ectopic DEF6 attributed to ~50% of the cell population, on the background of high endogenous levels. Endogenous DEF6 might thus mask potential effects of overexpressed mutant DEF6 constructs. Furthermore, the validation experiments in T cells only confirmed a reduced activity of overexpressed DEF6-2A as compared to DEF6-WT overexpression, and future studies need to further confirm the relevance of these phosphosites in T cell activation. For example, studies in T cells from DEF6 knockout mice or human DEF6 knockout T cell lines, combined with DEF6 phosphosite-mutant knockin or ectopic DEF6 mutant overexpression, could help to further confirm and elucidate the role of the DEF6 phosphosites in T cells.

Furthermore, despite method optimization, T cells were not in an ideal state after transfection considering stimulatability and viability. For this reason, further analyses involving flow sorting, studying interaction with other cells including Treg suppression assays or any long-term assays have limited feasibility, and hence conclusions about causative functional effects of DEF6 mutation in Treg-mediated suppression remain speculative at this stage. Nevertheless, we believe that our optimized validation in primary T cells with minimally affected viability and manipulation is highly relevant. Such an approach is rare owing to the difficulty for plasmid transfection into unstimulated primary T cells which is, however, necessary to enable the study of early TCR signaling without cell pre-activation. A second plausible explanation for the apparent contradiction in the effect size of DEF6 mutation might be context dependency of DEF6 effects not only on T cells versus non-T cell lines but also on different T cell subsets. We focused on Th1 cytokine expression here due to the relatively low expression of Th2 and Th17 cytokines in the cell population and time points studied here (data not shown). In this context, it is worth noting that IP_3_R-mediated Ca^2+^ release was shown to be needed for initial IL-2 and IFN-γ production while it inhibited IL-17 expression (64), and a differential role for DEF6 as IP_3_R regulator in this regard is conceivable. Consequently, it is plausible that Treg-mediated Ca^2+^/NFAT suppression may have diverse effects on expression of different effector cytokines, and interestingly—although Tregs are generally able to suppress Th1, Th2, and Th17 cytokines—Th17 cells seem refractory to Treg-mediated suppression in several reports (65–67). However, DEF6 not only affects Th1 and Th2 responses but also plays a role in murine Th17-mediated autoimmunity (45, 68). These findings further support a context dependency of Tregs applying different suppressive mechanisms depending also on the target cell type, and highlight the complexity of TCR signaling and Treg-mediated suppression.

In this context, it should be mentioned that we previously demonstrated that Tregs suppressed Ca^2+^ store depletion, NFAT, and NF-κB signaling in Tcons. Notably, artificial Ca^2+^ store depletion by suitable concentrations of ionomycin could abrogate suppression of both NFAT and NF-κB (and consequently of IL-2 expression), which suggested that Treg-mediated Ca^2+^ suppression was causative for both NFAT and NF-κB suppression (24). In light of the observation that both DEF6 and Tregs affected similar pathways (Ca^2+^ store depletion downstream of TCR-proximal events, PLCγ1, and IP_3_ generation), our current results suggest that Treg-mediated suppression of DEF6 phosphorylation and hence inhibition of DEF6–IP_3_R interaction may be causative for suppression of Ca^2+^ store depletion and downstream signaling. However, studies in Jurkat cells and DEF6^−/−^ murine CD4^+^ T cells showed that while DEF6 was required for Ca^2+^ store depletion, NFAT activation, and IL-2 production, it had only slight influences on the AP-1 pathway and was dispensable for NF-κB activation (16). This may be resolved by alternative explanations. First, compensatory mechanisms may confer full NF-κB activation in DEF6^−/−^ T cells. Similarly, alterations in signaling in the transformed Jurkat cell line may alter NF-κB signaling, since Jurkat cells are deficient in PTEN and SHIP-1, resulting in hyperactive AKT and ITK (69) both of which affect NF-κB and other TCR signaling pathways. Second, even though experimentally enhanced Ca^2+^ store depletion abrogated suppression of NFAT and NF-κB (24), it cannot be excluded that strong Ca^2+^ signaling overrides other unknown pathways that may be responsible for Treg-mediated NF-κB suppression. Indeed, NF-κB activation is complex and includes the major activation pathway through DAG, PKCθ, and the CBM complex, and also CD28 costimulation enhances NF-κB activation and there is cross talk with calcium signaling as well: Ca^2+^-dependent calcineurin activation can enhance NF-κB activation, and both positive and negative effects of Ca^2+^ on the NF-κB-activating CBM complex have been described (8). Furthermore, different readouts and time points in different studies to assess highly dynamic NFAT and NF-κB activity are difficult to compare. Finally, the mechanisms of Treg-mediated suppression and the influence of DEF6 may depend on the specific subset of Tregs and target cells, stimulation conditions, and timing (8).

With regard to Treg-mediated suppression of TCR signaling in Tcons, several studies have followed up our initial finding of Ca^2+^ suppression (24), yet no unbiased studies of suppressed T cell signaling have been performed until the present work. The other targeted studies on TCR signaling in Tcons have come to partially overlapping conclusions, supporting the concept of context dependency in Treg suppression mechanisms. While NF-κB suppression was consistently observed in several studies across human and mouse T cells and with different experimental setups (24, 26, 27), the results on Ca^2+^/NFAT suppression seem more diverse. It should be noted that, as in T cell activation, NFAT proteins seem to have a dual role in suppression: while Treg-mediated inhibition of NFAT reduces initial cytokine transcription, at later time points, NFAT itself has an inhibitory role as it is crucial to confer anergy. Accordingly, while we and others observed rapid inhibition of Ca^2+^/NFAT in Treg-suppressed Tcons (24, 25), NFAT nuclear translocation was not suppressed or even increased in suppressed cells at late time points (26–28, 70), although confirming our results, NFAT pathway inhibition occurred at early time points in the same studies (26). Along these lines, NFAT1 or NFAT1/4 knockout cells were less susceptible to suppression, possibly due to the inhibitory roles of NFAT in repressing cytokine expression (together with other factors such as inducible cAMP early repressor) and promoting anergy in long-term stimulated cells (28, 71). Furthermore, it is well known that Tregs need to be activated to be suppressive and particularly for short-term suppression assays, Treg pre-activation is crucial to observe rapid suppression of signaling (8). Those researchers who did not detect Ca^2+^/NFAT suppression also did not pre-activate Tregs (12, 27), although we and others have demonstrated that only pre-activated Tregs suppress Ca^2+^/NFAT signaling (24, 25). One should also note that it cannot be excluded that species, specific cell subset, and NFAT isoform expression may contribute to the observed differences. Regarding the lack of AP-1 pathway suppression, several studies are concordant (24, 27) at least when early-phase suppressive mechanisms were studied (26).

Along with DEF6, phosphorylation of several proteins that we have identified upon TCR stimulation and/or Treg-mediated suppression of Tcons open interesting avenues for revisiting the role of CD4^+^ T cells as targets for therapeutic intervention particularly in autoimmune diseases, and possibly in immunological and other malignancies as well. This is evident from the fact that DEF6 and several other molecules from our screen have been proposed or established as viable drug targets [unpublished observation from analysis with MetaCore Drug Lookup tool, and Otsubo et al. (72)]. Although aberrant expression may occur in tumor settings (72), the almost exclusive expression of DEF6 in T cells renders DEF6 as an especially interesting selective target for T cell-mediated inflammatory and autoimmune diseases. Since the phosphorylations covered in this study represent early events in T cell activation, they might be of interest to target causative events of immune system activation and suppression directly at the site of inflammation, where Tregs and Tcons interact. However, it is to be advised that phosphorylation and contact-dependent suppression of Tcons which we are focusing on in this study are one of the multiple mechanisms employed by Tregs depending on the immune micro-milieu and subsets of both Tregs and Tcons. This direct, rapid inhibition of Tcon signaling by Tregs might be relevant in the affected tissue to stop ongoing disease, as an additional means of suppression in case of failure of the mechanisms conferred by suppression of T cell priming in lymph nodes. We previously proposed that distinct suppressive mechanisms characterize suppression of T cell proliferation and priming predominantly *via* inhibition of APCs by Tregs, while direct Treg:Tcon interaction seems to promote suppression of cytokines (8). This notion is further supported by *in vivo* studies demonstrating direct Tcon:Treg interactions in the inflammatory tissue (13) but not in the lymph nodes (11, 12). Hence, the phosphorylation events revealed in our study may be key to understand the cause of resistance of Tcons toward suppression by Tregs, which has been reported in several autoimmune diseases (6, 7). In this context, our data provide novel targets for future exploration to modulate the sensitivity of responder T cells to suppression by Tregs, which is of interest for future therapeutic manipulation in autoimmune disease and cancer. Furthermore, several Treg-based immunotherapeutic clinical trials present an interesting prospect to study these phosphorylation events in the patients.

## Author Contributions

AS and RJ designed all experiments and analyses, performed and analyzed T cell experiments for phosphoproteomics, interpreted results, and wrote the paper; RJ, AS, and FM prepared figures; RJ designed, performed, and analyzed T cell validation experiments with the help of AS; RJ, NB, FM, and AS analyzed data; FM and RJ designed and performed bioinformatics analyses; NB designed and performed proteomics sample preparation, mass spectrometry, and analysis; AA, ZS, and AS designed DEF6 mutagenesis and provided reagents; ZS performed mutagenesis and coimmunoprecipitation; AH supervised proteomics experiments; JT supervised research and contributed to analysis design; AS designed the project and supervised research. All authors read and approved the final version of the manuscript.

## Conflict of Interest Statement

The authors declare that the research was conducted in the absence of any commercial or financial relationships that could be construed as a potential conflict of interest. The reviewer, RR, and handling editor declared their shared affiliation.
