# Supplementary material for: Phosphoproteomics Reveals Regulatory T Cell-Mediated DEF6 Dephosphorylation That Affects Cytokine Expression in Human Conventional T Cells
Source: Front Immunol. 2017 Sep 25;8:1163. doi: 10.3389/fimmu.2017.01163 (PMC5622166; doi:10.3389/fimmu.2017.01163)
Supplement: Supplementary file 4 [file Image_3.PDF]

Supplementary Material

Phosphoproteomics Reveals Regulatory T Cell-Mediated DEF6 Dephosphorylation That Affects Cytokine Expression in Human Conventional T Cells

Rubin N. Joshi, Nadine A. Binai, Francesco Marabita, Zhenhua Sui, Amnon Altman, Albert J.R. Heck, Jesper Tegnér and Angelika Schmidt*

*** Correspondence:** Angelika Schmidt: Schmidt_Angelika@outlook.com

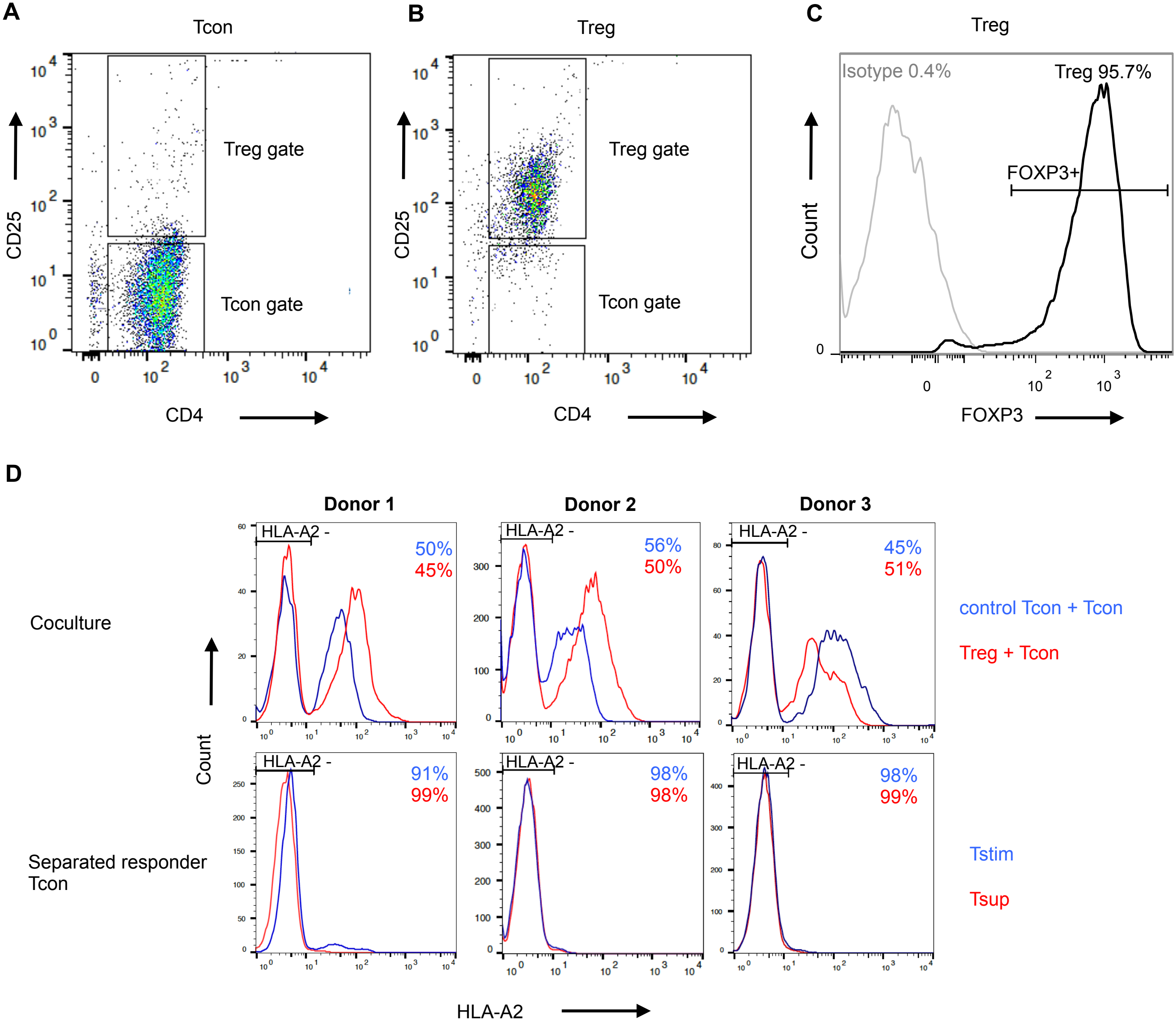

Figure S1. Quality control analysis of T cells used for phosphoproteomics.
Purity of isolated (**A**) Tcons and (**B, C**) Tregs. Tcons were isolated as $CD4^{+}CD25^{-}$ population from PBMCs. Tregs were isolated as $CD25^{++}$ population from PBMCs and the purity regarding CD4 and CD25 (surface staining) as well as intracellular FOXP3 staining is shown ("isotype": isotype control antibody for intracellular staining). Representative donors out of more than 20 donors each are shown. (**D**) Composition of cocultures and separated Tcons. Upper panel: A 1:1 cell ratio in cocultures of $HLA\text{-}A2^{-}$ responder Tcons with $HLA\text{-}A2^{+}$ Tregs or Tcons respectively was confirmed by flow cytometry, detecting the allogeneic marker HLA-A2 that was stained with anti-HLA-A2-FITC antibody. Lower panel: Purity of re-isolated $HLA\text{-}A2^{-}$ responder Tcons (Tstim and Tsup respectively) that were used for phosphoproteomics is represented after coculture separation. Percentage values given represent the fraction of $HLA\text{-}A2^{-}$ responder Tcons in cocultures before (upper panel) and after (lower panel) coculture separation for control Tcon:Tcon (blue) and Treg:Tcon (red) cocultures.

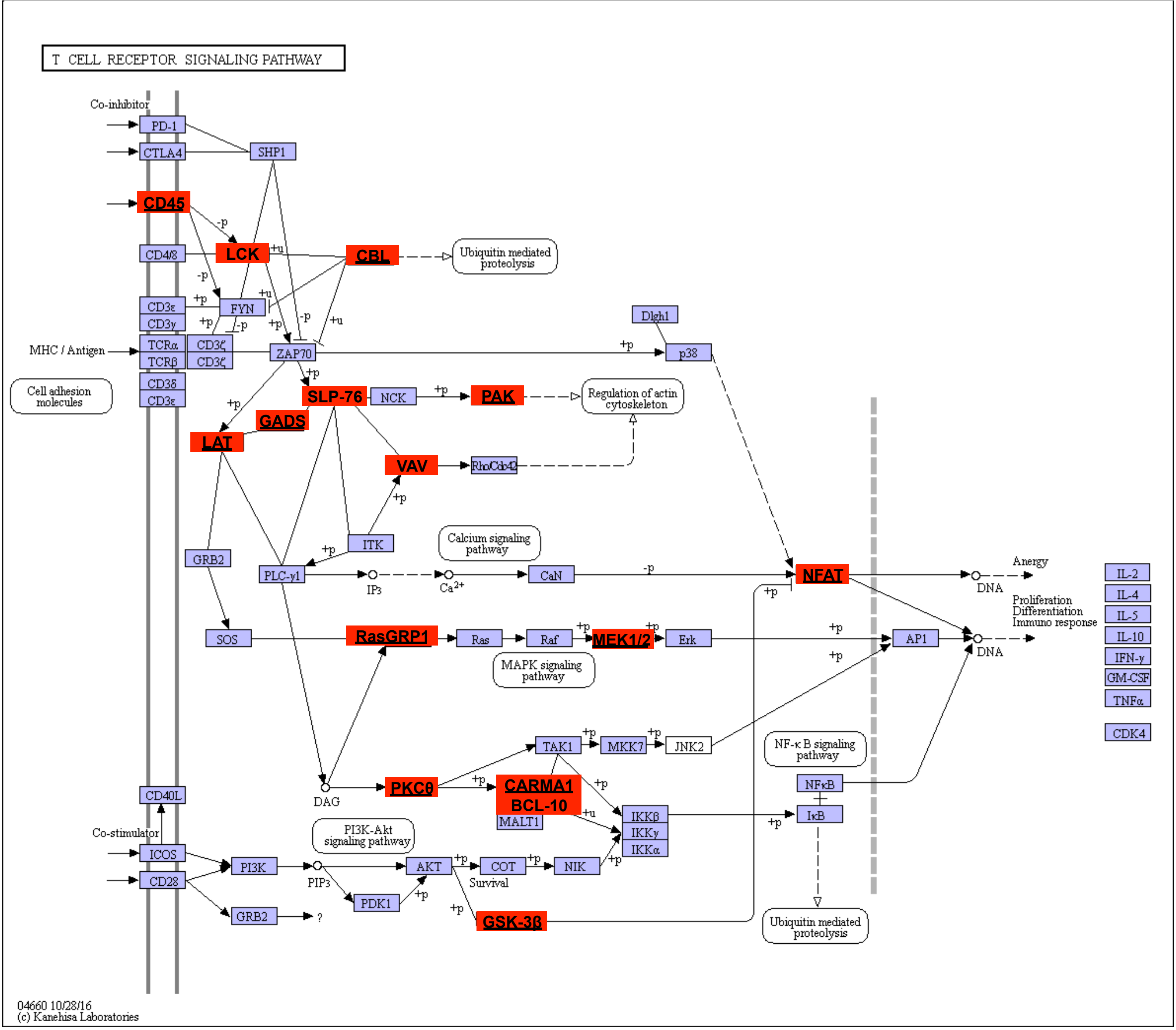

Figure S2. **Coverage of TCR pathway phosphoproteins detected in phosphoproteomics samples.** Proteins that are reported to be involved in TCR signaling according to the Kyoto Encyclopedia of Genes and Genomes (KEGG) database are shown in blue, and all proteins with phosphopeptides detected in at least one donor are marked in red while the ones detected in at least 2 of 3 donors (and hence quantified as in Supplementary Table S1) are further underlined. KEGG pathway image (T cell receptor signaling pathway, map04660) reproduced with permission from the copyright owner (Kanehisa Laboratories).

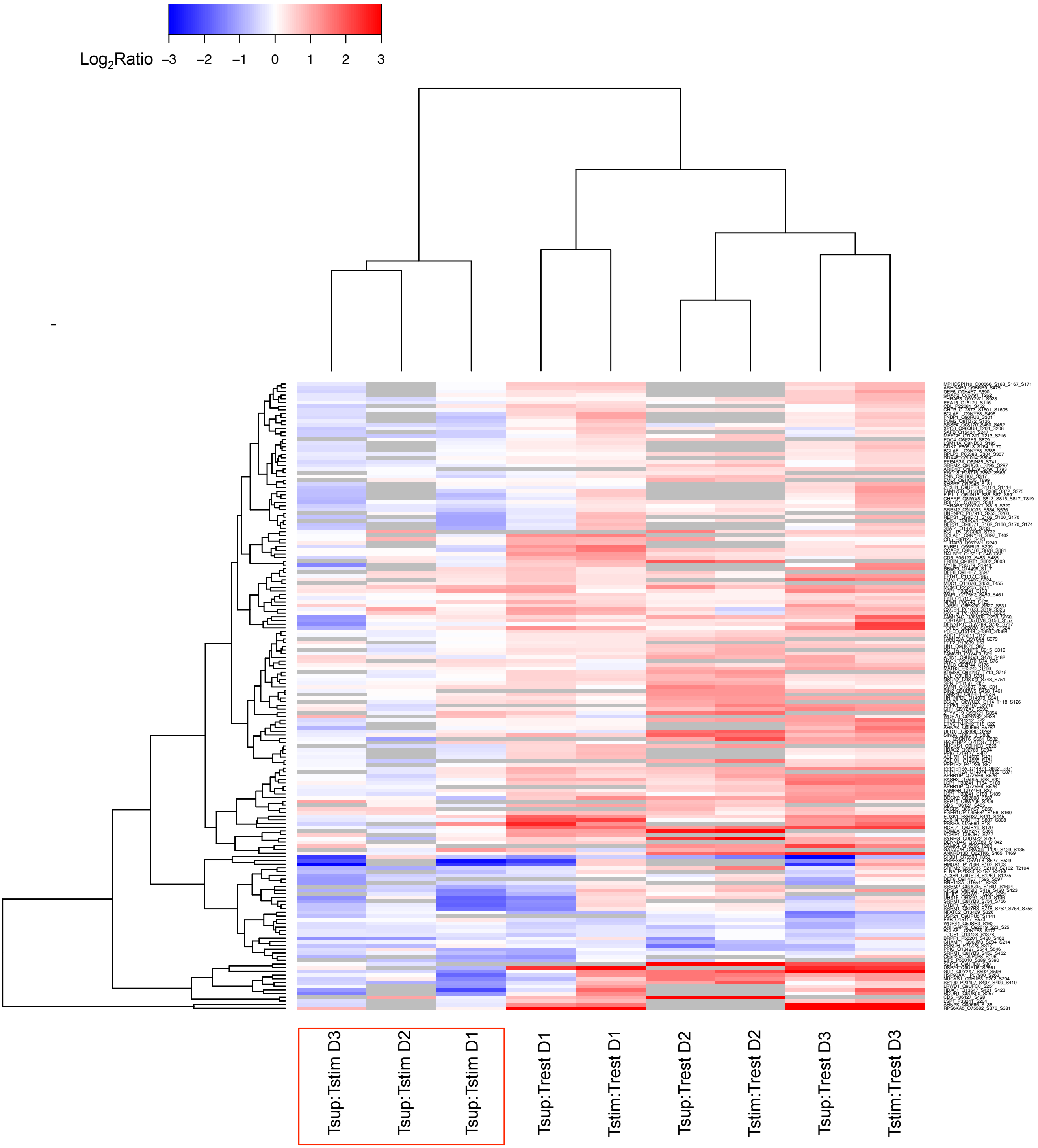

Figure S3. Tregs enforce a unique signature on the phosphoproteome on Tcons.
The heat map represents the $\log_2$ ratio in dimethyl intensities of phosphopeptides between indicated comparisons and donors ("D1", "D2", "D3" = Donor 1, 2 ,3). The phosphopeptides are represented by their corresponding gene names, protein accession number and localized phosphosite. Multiple listings of the same gene and phosphosite denote presence of multiple unique phosphopeptides. The magnitude of change is represented according to the indicated color scale. Missing values in individual donors are depicted in gray. Only phosphopeptides changing with $P < 0.05$ in any of the three ratios and detected in at least 2 out of 3 donors are included. Clustering indicates complete linkage Euclidian distance.

Donor	Gene	Uniprot Ac.	Sequence	Phosphosite	Log_2 Tsup:Tstim	Log_2 Tstim:Trest
DONOR2						
	DEF6	Q9H4E7	WGSQGNRTPSPNSNEQQK	S590_S597	-0.156	0.538
	DEF6	Q9H4E7	WGSQGNRTPSPNSNEQQK	S590_T595_S597	0.083	0.039
	DEF6	Q9H4E7	WGSQGNRTPSPNSNEQQK	S597	NA	0.098
	DEF6	Q9H4E7	WGSQGNRTPSPNSNEQQK	**T595_S597**	-0.439	0.436
DONOR3						
	DEF6	Q9H4E7	WGSQGNRTPSPNSNEQQK	S590_T595_S597	0.490	0.839
	DEF6	Q9H4E7	WGSQGNRTPSPNSNEQQK	**T595_S597**	-0.967	0.030
	DEF6	Q9H4E7	WGSQGNRTPSPNSNEQQK	**T595_S597**	-1.005	0.371
DONOR4						
	DEF6	Q9H4E7	WGSQGNRTPSPNSNEQQK	S590_S597	-0.219	0.703
	DEF6	Q9H4E7	WGSQGNRTPSPNSNEQQK	S590_T595_S597	-0.200	0.513
	DEF6	Q9H4E7	WGSQGNRTPSPNSNEQQK	S590_T595_S597	-0.509	0.495
	DEF6	Q9H4E7	WGSQGNRTPSPNSNEQQK	S597	NA	-0.023
	DEF6	Q9H4E7	WGSQGNRTPSPNSNEQQK	T595	-0.377	0.185
	DEF6	Q9H4E7	WGSQGNRTPSPNSNEQQK	T595	-0.091	0.434
	DEF6	Q9H4E7	WGSQGNRTPSPNSNEQQK	**T595_S597**	-0.377	0.185
	DEF6	Q9H4E7	WGSQGNRTPSPNSNEQQK	**T595_S597**	-0.091	0.434

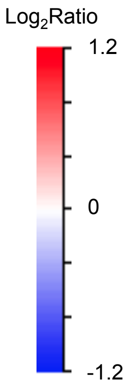

Figure S4. DEF6 T595_S597 phosphorylation in multiple phosphopeptides is changing upon activation and Treg-mediated suppression.

A fourth additional donor (“Donor4”) was processed for phosphoproteomics, but in contrast to donors 1, 2 and 3, was not used in summary and statistical analyses due to overall lower coverage and thus lower overlap of unique phosphopeptides with other donors. All DEF6 phosphopeptides containing pT595 and/or pS597 are listed, including the additional donor 4 (verification donor). The phosphopeptide of interest (phospho-T595_S597) is highlighted in bold letters. Values represent log_2 intensity ratios in the indicated comparisons for each donor, and are highlighted according to the indicated color scale. Raw values for dimethyl intensities without quantile normalization were used for the calculation of the displayed intensity ratios. Non-collapsed data are shown, and peptides listed twice represent detection in technical replicate MS runs; redundant peptides detected multiple times within the same run are listed only once. Note that in the main figures and for statistical analysis, collapsed and normalized intensity data of Donors 1, 2 and 3 were used, as described in the *Methods* section.

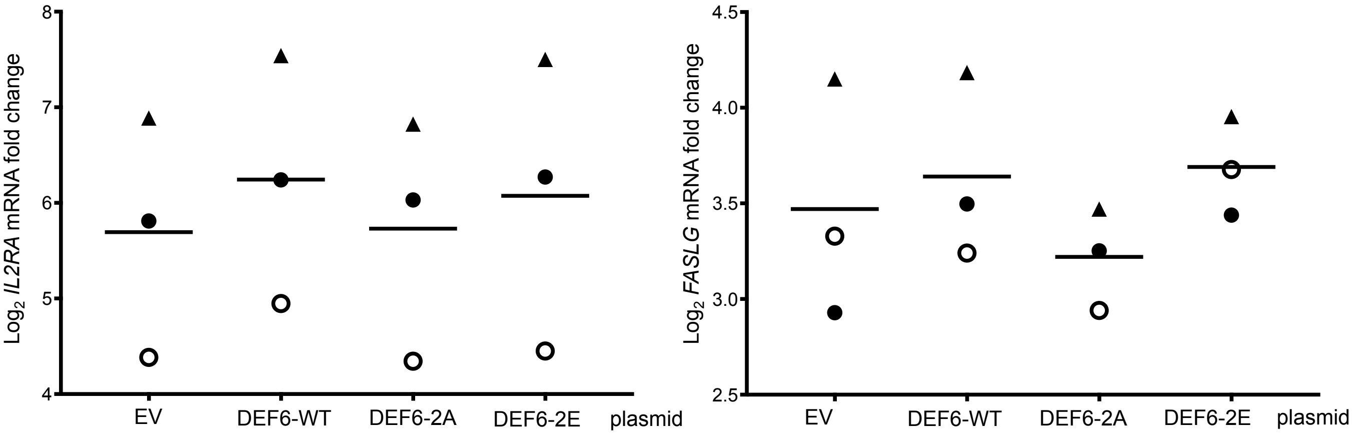

Figure S5. T595 and S597 phosphosites in DEF6 protein contribute to T cell activation.
Tcons transiently transfected with plasmids encoding the indicated DEF6 proteins or empty vector (EV) were treated as in Figure 5(D-E). The abundances of *IL2RA* and *FASLG* mRNA in transfected Tcons after 3 hours of stimulation were normalized to expression levels of *RPL13A* mRNA, unstimulated Tcons were set to 1, and fold change was calculated compared to unstimulated Tcons of the same donor. Results are presented as $\log_2$ fold change in the abundance of mRNA for individual donors (each symbol represents one donor; n=3 donors) along with mean values (lines). The donors are represented by the same symbol as in Figure 5(D-E).
